# Supplementary material for: Spatial distribution and determinants of micronutrient intake status among children aged 6–23 months in Ethiopia: A Multi-scale Geographical Weighted Regression Analysis
Source: PLoS One. 2025 Apr 29;20(4):e0321113. doi: 10.1371/journal.pone.0321113 (PMC12040264; doi:10.1371/journal.pone.0321113)
Supplement: Attachment — Submitted filename: PONE-D-24-22661 12-09-2024.pdf [file pone.0321113.s002.pdf]

PLOS ONE

Spatial distribution and determinants of micronutrient intake status among children aged 6-23 months in Ethiopia: A Multi-scale Geographical Weighted Regression Analysis

--Manuscript Draft--

**Manuscript Number:**	PONE-D-24-22661
**Article Type:**	Research Article
**Full Title:**	Spatial distribution and determinants of micronutrient intake status among children aged 6-23 months in Ethiopia: A Multi-scale Geographical Weighted Regression Analysis
**Short Title:**	Spatial distribution and determinants of micronutrient intake status among children in Ethiopia
**Corresponding Author:**	Alemu Birara Zemariam, MSc<br>Woldia University<br>Woldia, ETHIOPIA
**Keywords:**	Children, Ethiopia, Micronutrient intake, Multi-scale geographical weighted regression, spatial distribution
**Abstract:**	Introduction: Micronutrient (MN) deficiency is a major global health concern, especially in developing countries like Ethiopia. However, there is a lack of comprehensive monitoring and information regarding MN intake status. This study aimed to assess the spatial distribution and factors influencing the intake of MN-rich foods among children aged 6-23 months in Ethiopia.<br><br>Methods: This study utilized the Ethiopian 2016 Demographic and Health Survey dataset with 2562 weighted children. The Bernoulli model was applied using Kuldorff's SaTScan version 9.6 software. The spatial distributions of MN-rich food intake were visualized using ArcGIS pro version 3.0 software. Model comparison was conducted using log likelihood ratio and corrected Akakie Information Criteria. A multi-scale geographical weighted regression analysis was performed using MGWR version 2.0 software. A P-value threshold of less than 0.05 was used to identify spatially significant predictors.<br><br>Results: Overall, 69% (95% CI: 60.87, 77.43) of children aged 6–23 months in Ethiopia were found to have consumed foods rich in MN. The intake of these nutrient-rich foods exhibited significant clustering in specific regions, including Addis Ababa, Dire Dawa, Harari, certain parts of Benishangul, and the Gambella region. Spatial scan statistics analysis identified a total of 65 primary spatial clusters. Children residing within the primary clusters were found to be 29% more likely to have an intake of foods rich in MN compared to those living outside the identified clusters (RR =1.29, LLR = 25.34, P < 0.001). Key spatially significant predictors included higher household wealth status; children aged 13-23 months, the presence of antenatal care, and mothers with any job.<br><br>Conclusion: The consumption of foods rich in MN in Ethiopia displayed non-random spatial patterns. To tackle the problem of inadequate intake of these nutritious foods and address the burden of MN deficiency among children aged 6-23 months, it is essential for policymakers and health planners to implement targeted nutrition interventions in the identified areas and factors.
**Order of Authors:**	Alemu Birara Zemariam, MSc
	Addis Wondmagegn Alamaw, MSc
	Rediet Woldesenbet Molla, MSc
	Tesfaye Engdaw Habtie, MSc
	Molla Azmeraw Bizuayehu, MSc
	Ribka Nigatu Haile, MSc

Powered by Editorial Manager® and ProduXion Manager® from Aries Systems Corporation

	Tegene Atamenta Kitaw, MSc
	Biruk Beletew Abate, MSc
	Mollalign Birara Aligaz
**Additional Information:**	
**Question**	**Response**
**Financial Disclosure**<br><br>Enter a financial disclosure statement that describes the sources of funding for the work included in this submission. Review the submission guidelines for detailed requirements. View published research articles from *PLOS ONE* for specific examples.<br><br>This statement is required for submission and **will appear in the published article** if the submission is accepted. Please make sure it is accurate.<br><br>**Funded studies**<br>Enter a statement with the following details:<br>• Initials of the authors who received each award<br>• Grant numbers awarded to each author<br>• The full name of each funder<br>• URL of each funder website<br>• Did the sponsors or funders play any role in the study design, data collection and analysis, decision to publish, or preparation of the manuscript?<br><br>Did you receive funding for this work?	The author(s) received no specific funding for this work.
**Competing Interests**<br><br>Use the instructions below to enter a competing interest statement for this submission. On behalf of all authors, disclose any competing interests that could be perceived to bias this work—acknowledging all financial support and any other relevant financial or non-financial competing interests.<br><br>This statement is required for submission	The authors have declared that no competing interests exist.

and **will appear in the published article** if the submission is accepted. Please make sure it is accurate and that any funding sources listed in your Funding Information later in the submission form are also declared in your Financial Disclosure statement.<br><br>View published research articles from *PLOS ONE* for specific examples.<br><br>**NO authors have competing interests**<br><br>Enter: *The authors have declared that no competing interests exist.*<br><br>**Authors with competing interests**<br><br>Enter competing interest details beginning with this statement:<br><br>*I have read the journal's policy and the authors of this manuscript have the following competing interests: [insert competing interests here]*<br><br>* typeset	
**Ethics Statement**<br><br>Enter an ethics statement for this submission. This statement is required if the study involved:<br><br>• Human participants<br>• Human specimens or tissue<br>• Vertebrate animals or cephalopods<br>• Vertebrate embryos or tissues<br>• Field research<br><br>Write "N/A" if the submission does not require an ethics statement.<br><br>General guidance is provided below. Consult the submission guidelines for detailed instructions. **Make sure that all information entered here is included in the Methods section of the manuscript.**	The 2016 EDHS was ethically reviewed by the National Research Ethics Review Committee of the Ethiopian Ministry of Science and Technology. As described in the survey final report, involvement in the survey program was voluntary, and verbal agreement (informed consent) was also taken. The International Review Board of Demographic and Health Surveys program data archivists were allowed to download and use the datasets for this study. Also, the data was handled according to the Helsinki Declaration of the World Medical Association.

**Format for specific study types**<br><br>**Human Subject Research (involving human participants and/or tissue)**<br>• Give the name of the institutional review board or ethics committee that approved the study<br>• Include the approval number and/or a statement indicating approval of this research<br>• Indicate the form of consent obtained (written/oral) or the reason that consent was not obtained (e.g. the data were analyzed anonymously)<br><br>**Animal Research (involving vertebrate animals, embryos or tissues)**<br>• Provide the name of the Institutional Animal Care and Use Committee (IACUC) or other relevant ethics board that reviewed the study protocol, and indicate whether they approved this research or granted a formal waiver of ethical approval<br>• Include an approval number if one was obtained<br>• If the study involved *non-human primates*, add *additional details* about animal welfare and steps taken to ameliorate suffering<br>• If anesthesia, euthanasia, or any kind of animal sacrifice is part of the study, include briefly which substances and/or methods were applied<br><br>**Field Research**<br><br>Include the following details if this study involves the collection of plant, animal, or other materials from a natural setting:<br>• Field permit number<br>• Name of the institution or relevant body that granted permission	
**Data Availability**<br><br>Authors are required to make all data underlying the findings described fully available, without restriction, and from the time of publication. PLOS allows rare exceptions to address legal and ethical concerns. See the PLOS Data Policy and FAQ for detailed information.	Yes - all data are fully available without restriction

Powered by Editorial Manager® and ProduXion Manager® from Aries Systems Corporation

A Data Availability Statement describing where the data can be found is required at submission. Your answers to this question constitute the Data Availability Statement and **will be published in the article**, if accepted.<br><br>**Important:** Stating 'data available on request from the author' is not sufficient. If your data are only available upon request, select 'No' for the first question and explain your exceptional situation in the text box.<br><br>Do the authors confirm that all data underlying the findings described in their manuscript are fully available without restriction?	
**Describe where the data may be found in full sentences. If you are copying our sample text, replace any instances of XXX with the appropriate details.**<br><br>• If the data are **held or will be held in a public repository**, include URLs, accession numbers or DOIs. If this information will only be available after acceptance, indicate this by ticking the box below. For example: *All XXX files are available from the XXX database (accession number(s) XXX, XXX.*).<br>• If the data are all contained **within the manuscript and/or Supporting Information files**, enter the following: *All relevant data are within the manuscript and its Supporting Information files.*<br>• If neither of these applies but you are able to provide **details of access elsewhere**, with or without limitations, please do so. For example:<br><br>*Data cannot be shared publicly because of [XXX]. Data are available from the XXX Institutional Data Access / Ethics Committee (contact via XXX) for researchers who meet the criteria for access to confidential data.*<br><br>*The data underlying the results presented in the study are available from (include the name of the third party*	This study dataset used for the current study was publicly available in the DHS repository (https://dhsprogram.com).

and contact information or URL).<br>• This text is appropriate if the data are owned by a third party and authors do not have permission to share the data.<br><br>* typeset	
Additional data availability information:	

Powered by Editorial Manager® and ProduXion Manager® from Aries Systems Corporation

Manuscript
Click here to access/download;Manuscript;MN_manuscript V_1.docx

Spatial distribution and determinants of micronutrient intake status among children aged 6-23 months in Ethiopia: A Multi-scale Geographical Weighted Regression Analysis

Alemu Birara Zemariam[1*], Addis Wondmagegn Alamaw[2], Rediet Woldesenbet Molla[3], Tesfaye Engdaw Habtie[4], Molla Azmeraw Bizuayehu[1], Ribka Nigatu Haile[1], Tegene Atamenta Kitaw[4], Biruk Beletew Abate[1], Mollalign Aligaz[1]

[1] Department of Pediatrics and Child Health Nursing, School of Nursing, College of Medicine and Health Science, Woldia University, Woldia, Ethiopia

[2] Department of Emergency and Critical Care Nursing, School of Nursing, College of Medicine and Health Science, Woldia University, Woldia, Ethiopia

[3] Department of Midwifery, School of Midwifery, School of Midwifery, College of Medicine and Health Science, Woldia University, Woldia, Ethiopia

[4] Department of Nursing, School of Nursing, College of Medicine and Health Science, Woldia University, Woldia, Ethiopia

*Corresponding Author: Alemu Birara Zemariam, Woldia University, Woldia Ethiopia.

E-mail: alexb7298@gmail.com Tel: +251913162327

Abstract

Introduction: Micronutrient (MN) deficiency is a major global health concern, especially in developing countries like Ethiopia. However, there is a lack of comprehensive monitoring and information regarding MN intake status. This study aimed to assess the spatial distribution and factors influencing the intake of MN-rich foods among children aged 6-23 months in Ethiopia.

Methods: This study utilized the Ethiopian 2016 Demographic and Health Survey dataset with 2562 weighted children. The Bernoulli model was applied using Kuldorff's SaTScan version 9.6 software. The spatial distributions of MN-rich food intake were visualized using ArcGIS pro version 3.0 software. Model comparison was conducted using log likelihood ratio and corrected Akakie Information Criteria. A multi-scale geographical weighted regression analysis was performed using MGWR version 2.0 software. A P-value threshold of less than 0.05 was used to identify spatially significant predictors.

Results: Overall, 69% (95% CI: 60.87, 77.43) of children aged 6–23 months in Ethiopia were found to have consumed foods rich in MN. The intake of these nutrient-rich foods exhibited significant clustering in specific regions, including Addis Ababa, Dire Dawa, Harari, certain parts of Benishangul, and the Gambella region. Spatial scan statistics analysis identified a total of 65 primary spatial clusters. Children residing within the primary clusters were found to be 29% more likely to have an intake of foods rich in MN compared to those living outside the identified clusters (RR =1.29, LLR = 25.34, P < 0.001). Key spatially significant predictors included higher household wealth status; children aged 13-23 months, the presence of antenatal care, and mothers with any job.

Conclusion: The consumption of foods rich in MN in Ethiopia displayed non-random spatial patterns. To tackle the problem of inadequate intake of these nutritious foods and address the burden of MN deficiency among children aged 6-23 months, it is essential for policymakers and health planners to implement targeted nutrition interventions in the identified areas and factors.

Key words: Children, Ethiopia, Micronutrient intake, Multi-scale geographical weighted regression, spatial distribution

Introduction

Micronutrient (MN) deficiency is the global public health concern among children and it is worse in developing countries including Ethiopia [1-3]. Notably, MN during the first 1000 days of child life is critical for optimal growth, health and development, but little attention has been given to MN supplementation status [4]. Children, especially those in low and middle-income countries like Ethiopia, are particularly vulnerable to under nutrition [1]. Globally, malnutrition remains a significant issue, with one in three children under the age of five not receiving sufficient nutrition. MN deficiencies often go unnoticed, leading to around 340 million children worldwide experiencing what is termed "hidden hunger"[5]. Children need to receive adequate nutrient supplementation to support their growth and overall well-being. MN is basically classified into two groups like vitamins and minerals. Iron, zinc, iodine, vitamins like vitamin A and folic acid (vitamin B9) are the listed micro-nutrients associated with the cause of mortality and morbidity in children aged under 5 [6]. Despite being needed in smaller quantities, MN contribute significantly to the production of hormones, enzymes, and other essential substances necessary for growth and well-being [7].

In Ethiopia around 37.3% children aged 6-23 months does not get enough MN supplement according to their age [8]. Reports from national surveys indicate concerning levels of MN deficiencies among Ethiopian children. Around 38% of children aged 6-59 months in urban areas and 24% in rural areas suffer from either iron or folate deficiency, with iron deficiency being a primary cause of anemia, affecting 54% of children in this age group according to 2016 Ethiopian demographic and health survey (EDHS) report only 9.24% of children take iron [9, 10]. Additionally, vitamin A deficiency alone is responsible for 80,000 deaths among preschool children in Ethiopia, with only 63% of children nationwide receiving adequate supplementation which is far from the government plan which is 90%. Furthermore, approximately 35% of Ethiopian children suffer from zinc deficiency, despite its crucial role in immune system function and overall well-being [11, 12].

Various nations employ different strategies to address MN deficiency and enhance their intake in children, including supplementation, fortification, food diversification, and public health interventions. The World Health Organization recommends MN supplementation for children due to the significant impact of deficiency on their well-being, providing guidelines to support

these efforts [6, 13]. Studies support the effectiveness of supplementation programs in addressing MN deficiency, either through individual supplementation of specific micronutrients like zinc, iodine, iron, or vitamin A, or through multiple MN supplementation combining three or more micronutrients in a single dose [14].

Parental educational status, maternal occupational status, ANC follow up of the mother, working status, birth weight of the child, wealth index, residency area, geography and racial differences of the country are listed among the factors which are associated with MN intake [8, 15]. Most studies on MN intake status in Ethiopia are confined to specific districts or regions and the geospatial distribution of MN intake status among children in Ethiopia remains poorly understood, hindering the development of geographically targeted interventions. Furthermore, data on hot and cold spot areas for MN intake is scarce. To expedite progress towards improving child health and ending child malnutrition, understanding the geospatial distribution of MN intake and its determinants among children is crucial. Thus, this study aims to better understand the spatial pattern of MN intake status and its determinants among children aged 6-23 months in Ethiopia using the nationally representative data.

Methods

Study setting, data source, and study period

We utilized the 2016 EDHS, which was a cross-sectional data in nine geographical region namely Tigray, Afar, Amhara, Oromia, Somalia, Benishangul-Gumuz, Southern Nation Nationality, and People's Region (SNNPR), Gambella, and Harari and two administrative cities (Addis Ababa and Dire Dawa).

We obtained permission and ethical approval from DHS data archivist to extract the data from www.measuredhs.com. The EDHS surveys are carried out roughly every 5 years aimed at generating updated health and health-related indicators. Sample weights were applied to ensure the representativeness of the samples and account for the complex survey design, survey non-response, and post-stratification. A multistage stratified sampling technique was used. The study focused on children aged 6-23 months using the kid’s records (KR) file. After performing data cleaning and exploration, our analysis included a total weighted sample of 2562 children aged 6-23 months with a total of 21 features [16].

Source and study population

The target population for this study consisted of all living children aged 6-23 months with their mothers in Ethiopia whereas, all children aged 6-23 months in the selected enumeration area were the study population.

Study variables and measurements

The outcome of interest was the status of micronutrient intake. **Micronutrient intake** was assessed by taking into account different elements, including the consumption of foods rich in Vitamin A or iron within the last 24 hours, the use of multiple micronutrient powder (MNP) or iron supplements in the preceding 7 days, and the administration of vitamin A supplements or deworming treatment within the past 6 months [17]. Consequently, if the respondent reported that the child had eaten' at least one of the minimum recommended MNs, we considered it "Yes"; if the children received none of the minimum recommended MNs, it was considered as "No".

To assess the consumption of foods rich in vitamin A (VA), we analyzed the intake of seven specific food groups within the previous 24 hours. These food groups included eggs, various meats (such as beef, pork, lamb, and chicken), pumpkin, carrots, and squash, dark green leafy vegetables, mangoes, papayas, and other fruits rich in VA, as well as liver, heart, and other organs, and fish or shellfish. Similarly, we assessed the consumption of iron-rich foods by examining the intake of four specific food groups within the previous 24 hours. These food groups consisted of eggs, various meats, liver, heart, and other organs, as well as fish or shellfish. To determine the intake of MNP, we asked the respondents if their child had received such powders in the past seven days. For assessing iron supplementation, we inquired whether the child had been given iron pills, sprinkles with iron, or iron syrup within the past seven days. The researchers examined vitamin A supplementation (VAS) and deworming treatment by reviewing the integrated child health card, which contains information on immunization and growth monitoring history. They also obtained verbal responses from the mothers. These assessments were specifically conducted for children aged 6-23 months to determine if they had received VAS and deworming treatment in the last six months. If the respondent reported that the child had consumed at least one of these food groups, it was categorized as a "Yes" response, indicating the consumption of MN-rich foods.

This study considered different independent variables to identify determinants of micronutrient intake among children aged 6-23 months in Ethiopia (Table 1).

Table 1:List of independent variables for the assessment of spatial distribution of micronutrient intake and its predictors among children aged 6-23 months in Ethiopia using 2016 EDHS.

Variable	Descriptions (classification)
Sex of the child	Male or female
Age of the child	6-12 month, 13-23 month
Mother age	15–24, 25–34, above 35 years
Residence status	Urban or Rural
Region	Tigray, Amhara, Oromia, SNNPR, Benishangul, Afar, Gambela, Somali, Harari, Addis Ababa, and Dire Dawa
Mother educational status	No education, Primary, Secondary, and Higher
Mothers working status	Working or not working
Wealth index	Poor, Middle, and Rich
Desire more children	No more want, undecided, wants more
Antenatal follow-up	No or Yes
Place of delivery	Home or health institution
Postnatal care	No or Yes
Family size	Less than five and greater or equal to five
Sex of household	Male or Female
Media access/ exposure	Yes or No
Had cough	Yes or No
Family size	< 5 and 5 or above
Had diarrhea	Yes or No

Data processing and analysis

The data was obtained from the EDHS 2016 dataset, and STATA version 17 was employed to perform non-spatial analysis such as calculating descriptive statistics using frequency and percentage. The data underwent processes like weighting, cleaning, editing, and recoding. For mapping the MN intake status at regional levels and identify spatially significant predictors, ArcGIS Pro version 3.0, Sat Scan version 9.6, and MGWR version 2.0 were employed.

Spatial autocorrelation and hot spot analysis

The Global Moran's I statistic was used to evaluate the regional variation of micronutrient intake among children aged 6-23 months in Ethiopia. When the Moran's I values are close to -1, it indicates that the consumption of micronutrient is poor and dispersed. On the other hand, values close to +1 indicate that the consumption is good and clustered. A Moran's I value of zero suggests that the consumption is randomly distributed across regions. Spatial autocorrelation is declared at a statistically significant Moran's I p-value less than 0.05 [18]. Additionally, there is a type of autocorrelation known as incremental autocorrelation that occurs at different distances. Z-scores for spatial autocorrelation were calculated at various distances to assess the strength and statistical significance of spatial clustering. The highest Z-scores indicate the distances at which spatial grouping is most pronounced [19].

Additionally, the Hot Spot Analysis was conducted using Getis-Ord Gi* Spatial Statistics in order to identify spatial clusters that are statistically significant in terms of having either low (cold spots) or high (hot spots) levels of micronutrient intake. Clusters with high Gi* values indicate significant hot spots, whereas clusters with low Gi* values indicate significant cold spots.

Spatial interpolation

Ordinary Kriging interpolation technique was utilized to predict the MN intake status among children aged 6-23 months in the unsampled enumeration areas based on the tested population. This approach relaxes the assumption of a Gaussian distribution of the observed semi-variogram in the input data, which is often not realistic in practice. This interpolation method generates a new simulated semi-variogram at each location, taking into account the estimated semi-variogram from the input data. The weight of the new simulated semi-variogram is calculated using Bayes' rule [20].

Spatial scan statistics

We utilized a spatial scan statistics model based on Bernoulli distribution to identify the geographic areas where there are statistically significant clusters of micronutrient intake among children aged 6-23 months. This analysis was conducted using Kuldorff's SaTScan version 9.6 software [21]. A moving scanning window was employed to assess the distribution of micronutrient intake among children aged 6-23 months in the study area. The scanning window

was circular in shape and the identification of the most likely clusters was determined using P-values and log-likelihood ratio tests based on 999 Monte Carlo replications.

Spatial regression analysis

Ordinary least squares (OLS) regression

Once the hot spot areas were identified, spatial regression modeling was conducted to explore the factors influencing the observed spatial clustering. Initially, OLS regression analysis was fitted. The OLS model is a global model that estimates a single coefficient for each explanatory variable across the entire study area. Global models assume that the factors affecting micronutrient intake are consistent and do not vary geographically. However, this assumption of geographical independence may introduce bias into the parameter estimates. To assess the assumption of spatial dependency, the Koenker (BP) statistics was used which were statistically significant ($P<0.001$), indicated that the relationships were not consistent, possibly due to non-stationary or heteroscedasticity.

In the presence of spatial dependency, the coefficient of the independent variable varies locally, meaning that the predictor variables may or may not be significant in different local areas [22]. Therefore, it suggested further regression model to account their huge limitations such as GWR and MGWR.

Geographically weighted regression (GWR)

The variation in the relationship between variables across might not be the same across different clusters and it can be identified using GWR. Unlike OLS regression, which fits a single linear regression equation to all the data in the study area, GWR creates a separate equation for each cluster in the DHS data. OLS regression calibrates the equation using data from all features or clusters, whereas GWR uses data from nearby features or clusters. As a result, the coefficient in GWR takes on different values for each cluster [23]. To explore the varying relationships between micronutrient intake and explanatory variables in different geographic areas, a newly emerging geographic regression model called Multi-scale geographic weighted regression (MGWR) was employed. Unlike the traditional GWR model, MGWR not only allows coefficients to vary across space but also accommodates variations in scale among different covariates. MGWR takes into account different spatial scales by considering distinct neighborhoods for each explanatory variable, thereby capturing the complex spatial relationships between variables [24]. To assess the presence of multicollinearity between variables, the

Variance Inflation Factor (VIF) was calculated. A VIF value exceeding 7.5 indicates the existence of multicollinearity [25]. In order to select the most appropriate model, the Corrected Akakie Information Criterion (AICc) and adjusted R2 values were computed. The model with the lowest AICc and the highest adjusted R2 was considered the best-fitting model [26]. In all statistical analyses, a P-value threshold of less than 0.05 was used to determine statistical significance.

Results

Socio-demographic characteristics of the study participants

A total weighted of 2562 children aged 6–23 months were included in this study. More than one-third (34.5%) of the female children were had taken foods rich in micronutrient. Of the total, 1062 (41.5%) of children whose age 13-23 months had taken foods rich in micronutrient. The mean ± SD age of the children was 11.92 ± 4.27 months. More than half (52.3%) of the child mothers were in the age group of 25–34 years. Nearly half (45.0%) of the households had poor wealth status and majority (75.5%) of the respondents were reside in rural area. Regarding maternal health service utilization, more than two-third (75.1%) of respondents were had ANC follow-up and nearly half (48.5%) of the respondents were gave birth at health facility (Table 2).

Table-2: Socio-demographic characteristics of study participants 2016 EDHS.

Variables	Categories	Micronutrient intake status				Total Weighted (n=2562)	
		No		Yes			
		Count	Percent	Count	Percent	Count	Percent
Respondent's current age	15-24	242	9.4%	520	20.3%	762	29.4%
	25-34	390	15.2%	925	36.1%	1315	52.3%
	>=35	162	6.3%	323	12.6%	485	18.3%
Region	Tigray	51	2.0%	227	8.9%	278	12.8%
	Afar	120	4.7%	124	4.8%	244	7.0%
	Amhara	99	3.9%	130	5.1%	229	7.4%
	Oromia	126	4.9%	238	9.3%	364	13.5%
	Somali	151	5.9%	166	6.5%	317	9.4%
	Benishangul	29	1.1%	176	6.9%	205	10.0%
	SNNPR	86	3.4%	241	9.4%	327	13.6%
	Gambella	40	1.6%	127	5.0%	167	7.2%
	Harari	42	1.6%	114	4.4%	156	6.4%
	Addis Ababa	26	1.0%	108	4.2%	134	6.1%
	Dire Dawa	24	0.9%	117	4.6%	141	6.6%
Residence	Urban	98	3.8%	434	16.9%	532	24.5%
	Rural	696	27.2%	1334	52.1%	2030	75.5%
Respondent’s highest educational level	No education	568	22.2%	955	37.3%	1523	54.0%
	Primary	168	6.6%	545	21.3%	713	30.8%
	Secondary	36	1.4%	171	6.7%	207	9.7%
	Higher	22	0.9%	97	3.8%	119	5.5%
Religion	Orthodox	189	7.4%	571	22.3%	760	32.3%
	Muslim	470	18.3%	809	31.6%	1279	45.8%
	Protestant	120	4.7%	340	13.3%	460	19.2%
	Others**	15	0.6%	48	1.9%	63	2.7%
Family size	<5	260	10.1%	583	22.8%	843	33.0%
	>=5	534	20.8%	1185	46.3%	1719	67.0%
Sex of household head	Male	622	24.3%	1454	56.8%	2076	82.2%
	Female	172	6.7%	314	12.3%	486	17.8%
Wealth index combined	Poor	503	19.6%	795	31.0%	1298	45.0%
	Medium	92	3.6%	284	11.1%	376	16.1%
	Rich	199	7.8%	689	26.9%	888	39.0%

Currently pregnant	No or unsure	737	28.8%	1655	64.6%	2392	93.6%
	Yes	57	2.2%	113	4.4%	170	6.4%
Desire for more children	Wants more	567	22.1%	1214	47.4%	1781	68.7%
	Undecided	34	1.3%	80	3.1%	114	4.5%
	Wants no more	193	7.5%	474	18.5%	667	26.8%
Father’s education level	No education	446	17.4%	691	27.0%	1137	39.1%
	Primary	220	8.6%	642	25.1%	862	36.3%
	Secondary	70	2.7%	241	9.4%	311	13.6%
	Higher	58	2.3%	194	7.6%	252	11.0%
Father’s occupation	Not working	153	6.0%	304	11.9%	457	17.2%
	Working	641	25.0%	1464	57.1%	2105	82.8%
Respondent's occupation	Not working	565	22.1%	1005	39.2%	1570	56.8%
	Working	229	8.9%	763	29.8%	992	43.2%
Sex of child	Male	390	15.2%	885	34.5%	1275	50.1%
	Female	404	15.8%	883	34.5%	1287	49.9%
Age of child in months	6-12	422	16.5%	706	27.6%	1128	39.9%
	13-23	372	14.5%	1062	41.5%	1434	60.1%
ANC follow-up	No	364	14.2%	441	17.2%	805	24.9%
	Yes	430	16.8%	1327	51.8%	1757	75.1%
Place of delivery	Home	560	21.9%	911	35.6%	1471	51.5%
	Health facility	234	9.1%	857	33.5%	1091	48.5%
PNC check-up	No	754	29.4%	1580	61.7%	2334	89.4%
	Yes	40	1.6%	188	7.3%	228	10.6%
Had diarrhea recently	No	667	26.0%	1442	56.3%	2109	81.6%
	Yes	127	5.0%	326	12.7%	453	18.4%
Had cough in last two weeks	No	631	24.6%	1416	55.3%	2047	80.1%
	Yes	163	6.4%	352	13.7%	515	19.9%
Media exposure	No	588	23.0%	1069	41.7%	1657	60.5%
	Yes	206	8.0%	699	27.3%	905	39.5%

Note: others*-catholic, traditional, ANC-antenatal care, PNC-postnatal care

Spatial distribution of micronutrient intake status among children aged 6-23 months

The prevalence of micronutrient intake among children aged 6-23 months was 69.01 % (95% CI: 60.87%, 77.43%). The highest prevalence of micronutrient intake among children aged 6-23 months was observed in Tigray, SNNPR, Oromia, Gambella, and Benishangul Gumuz regions (Figure 1).

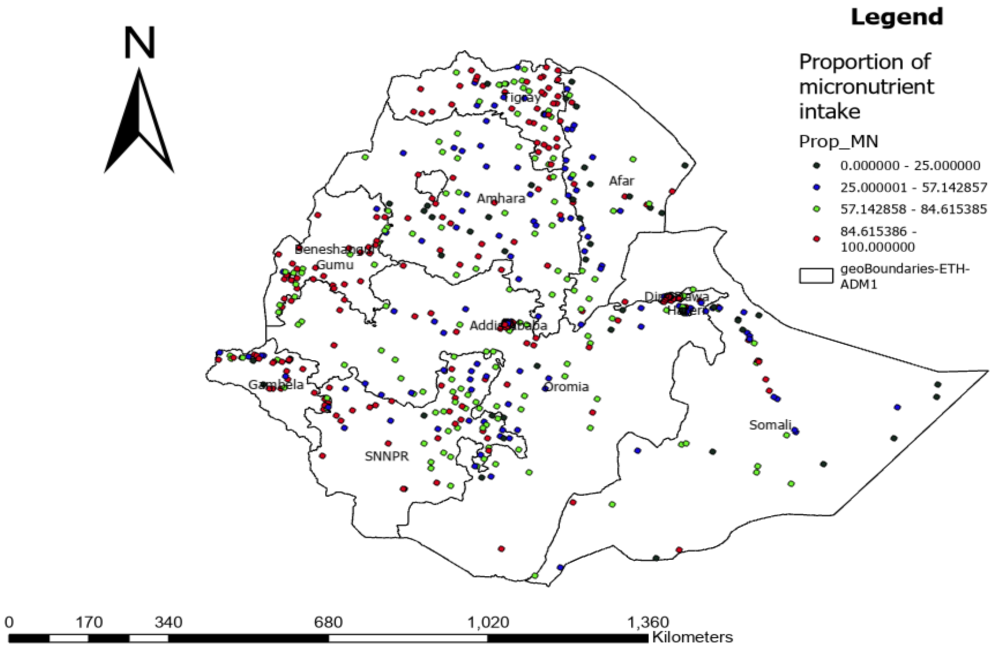

Source: Shape file from Central Stastics Agency in Ethiopia, 2013

Fig 1: Spatial distribution of micronutrient intake among children aged 6-23 months in Ethiopia 2016.

Spatial and incremental autocorrelation analysis

The spatial distribution of micronutrient intake among children aged 6-23 months in Ethiopia is clustered with Global Moran's I value of 0.167, p-value <0.001, and Z-score of 6.17. Thus, micronutrient supplementation status has a spatial dependency. In addition, the likelihood of this clustered pattern because of random chance is less than 1% (Figure 2)**.** The line graph of incremental autocorrelation shows the minimum and the maximum distance band. The minimum distance at the beginning was 96847.00 meters (Z-score= 7.52, P-value<0.001), whereas the first maximum peak was 184351.60 meters (Z-score= 11.05, P-value<0.001) (Figure 3)**.**

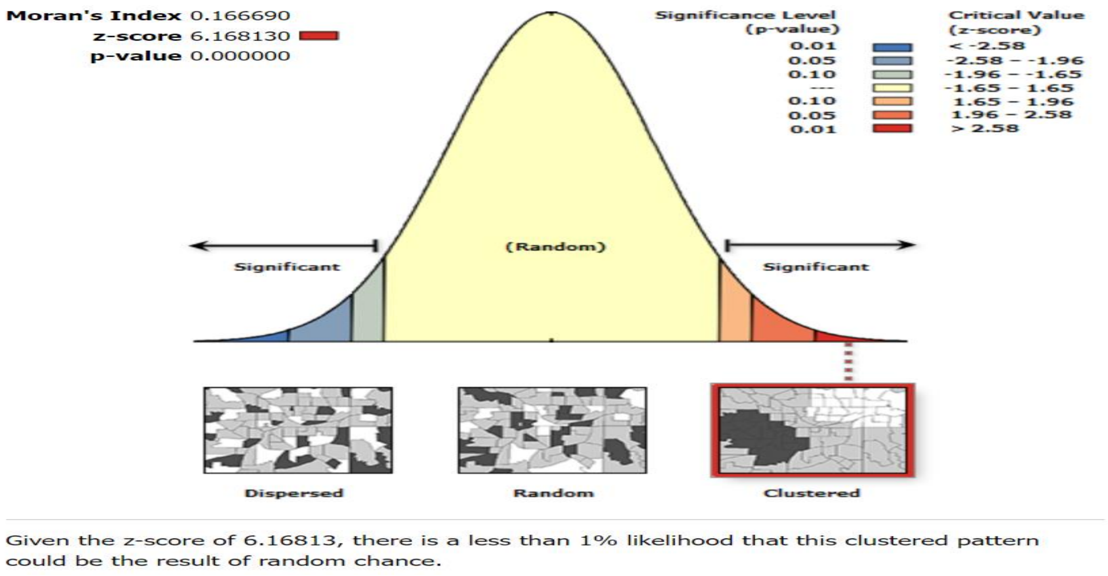

Fig 2: Spatial autocorrelation analysis of spatial distribution of micronutrient intake status among children in Ethiopia, 2016 EDHS, (weighted n= 2562).

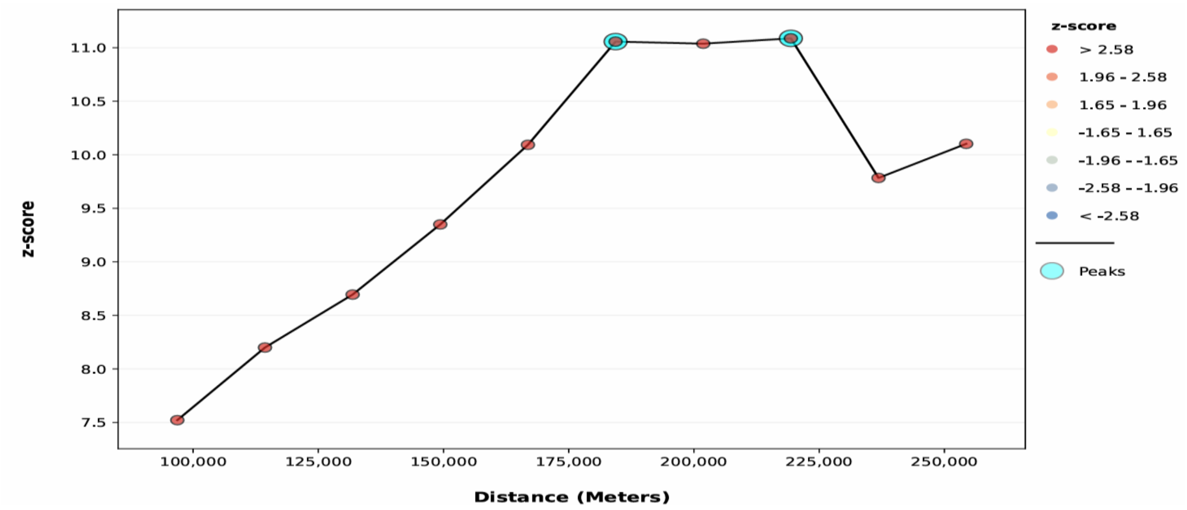

Fig 3: Incremental autocorrelation analysis of spatial distribution of micronutrient intake status among children in Ethiopia, 2016 EDHS, (weighted n= 2562).

Hot spot analysis

Hotspot and cold spot analysis was computed to identify areas with a high and low prevalence of micronutrient supplementation. Thus, significant clustering of micronutrient intake is detected in Addis Ababa, Harari, Dire Dawa, some parts of Gambella, and Benishangul Gumuz regions (Figure 4).

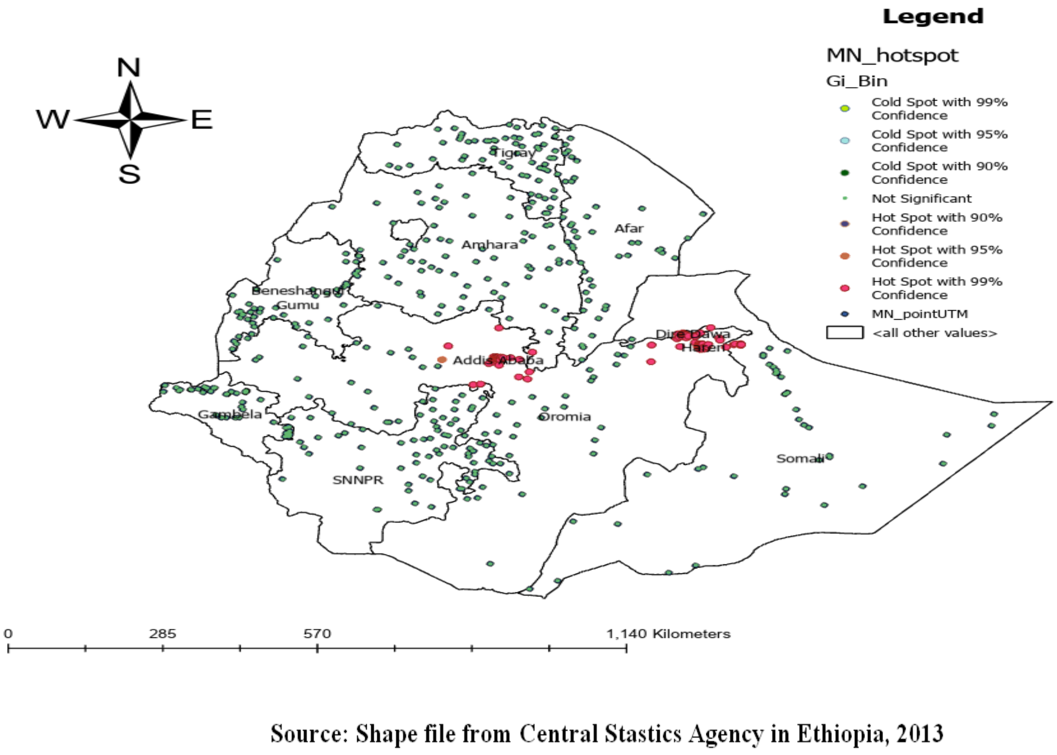

Fig 4: The Getis Ord Gi statistical analysis of hot spots of micronutrient intake among children in Ethiopia, EDHS 2016, (weighted n= 2562).

Spatial interpolation

Ordinary kriging interpolation was computed to predict the micronutrient intake distribution among children in Ethiopia. Thus, the highest predictive prevalence of micronutrient intake increased in the Dire Dawa, Harari, Addis Ababa, Somali, Gambella, some part of Afar regions. Meanwhile, low prediction was found in the remaining areas of Ethiopia (Figure 5**).**

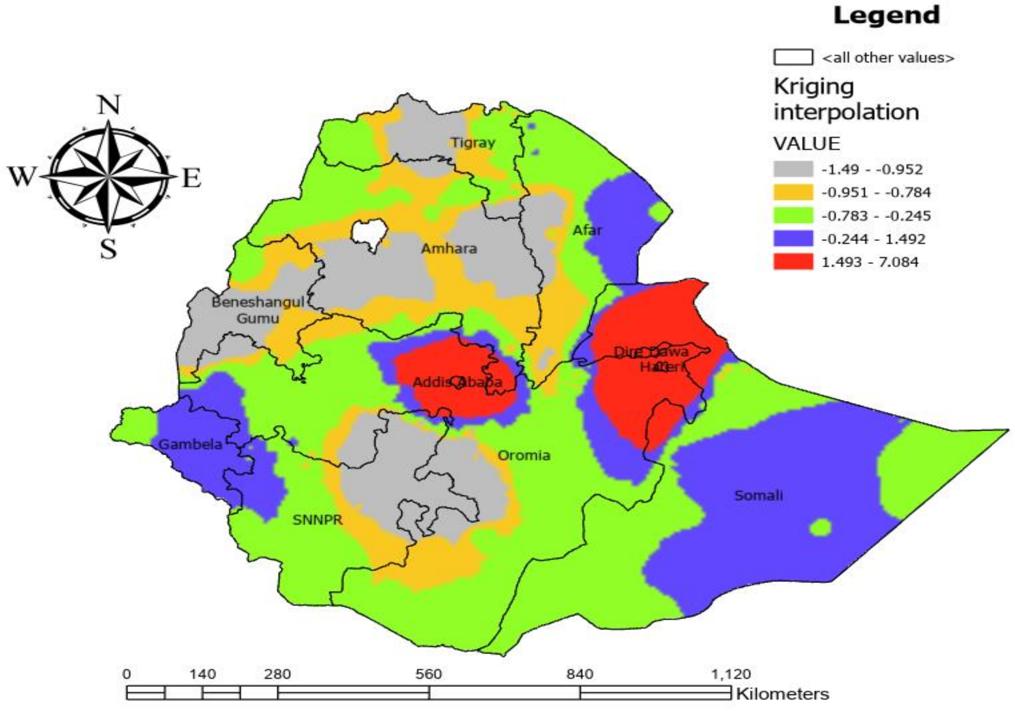

Source: Shape file from Central Stastics Agency in Ethiopia, 2013

Fig 5: Ordinary kriging interpolation to predict the prevalence of micronutrient intake among children in Ethiopia, 2016 EDHS (weighted n= 2562).

Sat scan stastical analysis

Purely spatial analysis using the Bernoulli model was done to identify clusters with high or low micronutrient intake rates. A total of 388 significant clusters (with in six spatial windows) were identified out of the total 612 imported clusters. Among the significant clusters, 65 clusters most likely (primary), and 323 clusters were secondary. The primary clusters were located at 9.759682 N, 35.443765 E in a 203.61 km radius in Addis Ababa, Oromia, SNNPR, Gambella and some part of Benishangul Gumuz region (Figure 6). Children were aged 6–23 months living in the primary cluster were 29 % more likely to take foods rich in micronutrient than outside the window (RR=1.29, LLR=25.34, P-value<0.001) (Table 3).

Table 3: Significant spatial scan statistics clusters of micronutrient intake among children aged 6–23 months, EDHS, 2016.

Cluster type	Detected cluster	Coordinate/Radius	Populatio	Cases	RR	LLR	P-value
Most likely cluster	621, 124, 88, 349, 65, 335, 304, 320, 569, 17, 563, 416, 209, 433, 374, 244, 407, 6, 595, 462, 184, 137, 183, 203, 175, 317, 409, 165, 395, 581, 150, 248, 508, 161, 193, 275, 457, 36, 643, 558, 559, 294, 285, 246, 533, 494, 498, 615, 411, 256, 555,	(9.759682 N, 35.443765 E) / 203.61 km	269	234	1.29	25.34	0.000000027**
1st Secondary	604, 481, 355, 461, 226, 45, 129, 84, 598, 341, 404, 430, 81, 89, 636, 590, 579, 156, 623, 575, 196, 220, 479, 400, 99, 94, 298, 237, 550, 117, 597, 192, 413, 605, 538, 384, 103, 424	(13.989017 N, 39.178052E) / 77.13 km	158	135	1.30	14.62	0.00037
2nd secondary cluster	140, 546, 363, 467, 644, 43, 606, 224, 385, 390, 111, 282, 27, 493, 185, 444, 514, 5, 631, 471, 74, 380, 613, 352, 202, 25, 30, 311, 594, 166, 557, 441, 281, 523, 242, 642, 473, 453, 610, 383, 329, 173, 443, 495, 238, 381, 393, 288, 60, 614, 396, 607, 28, 228, 56, 397, 157, 419, 357, 179, 257,	(9.624260 N, 41.839276 E) / 52.60 km	275	222	1.27	17.05	0.000026

3rd secondary cluster	180, 20, 141, 53, 434, 162, 126, 565, 347, 113, 505, 406, 503, 306, 388, 373, 360, 408, 215, 148, 41, 450, 216, 634, 609, 32, 420, 331, 445, 537, 600, 86, 232, 308, 574, 182, 272, 227, 297, 12, 502, 313, 447, 576, 486, 578, 468, 365, 577, 338, 342, 316, 470, 50, 21, 405, 271, 76, 398, 589, 142, 14, 518, 174, 359, 633, 34, 432, 26, 87, 154, 489, 522, 466, 204, 207, 262, 619, 54, 422, 62, 139, 477, 438, 586, 217, 376, 325, 562, 118, 23, 177, 437, 524, 213, 485, 168, 371, 552, 459, 234, 243, 299, 290, 554, 147, 526, 197, 119, 353, 83, 46, 149, 337, 261, 475, 145, 608, 123, 645, 539, 451, 225, 110, 487, 61, 59, 195, 399, 293, 31, 107, 635, 339, 19, 108, 11, 626, 330, 305, 414, 369, 211, 582, 155, 639, 15, 274, 532, 170, 428, 247, 153, 463, 509, 125, 560, 144, 112, 464, 402, 517, 287, 90, 319, 326, 303, 601, 289, 40, 472, 377, 452, 121, 529, 423, 219, 446, 245, 593, 270, 265, 122, 218, 71, 284, 394, 572, 350, 82, 417, 201, 531, 105, 510, 7, 51, 343, 567	(6.720108 N, 37.624880 E) / 403.23 km	818	598	1.32	30.73	0.0000000034
4th secondary cluster	253, 504, 612, 296, 258, 583, 268, 78, 98, 255, 528	(14.033877 N, 37.105922 E) / 118.26 km	54	48	1.67	15.24	0.000065
5th secondary cluster	143, 449, 392, 136, 128, 79	(12.830425 N, 39.451846 E) / 30.51 km	14	14	1.91	8.94	0.03

Note: RR-relative risk, LLR-log likelihood ratio, N-north, E-east

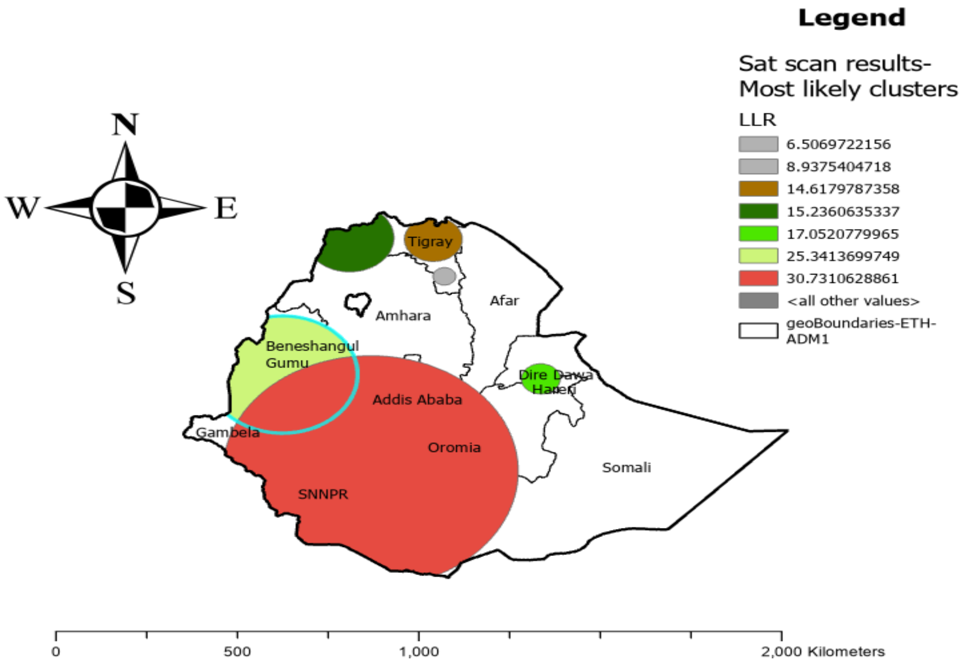

Source: Shape file from Central Stastics Agency in Ethiopia, 2013

Fig 6: SaTScan scan statistics of micronutrient intake among children in Ethiopia, 2016 EDHS (weighted n= 2562).

Ordinary least squares (OLS) regression analysis

An ordinary least squares model was computed to identify spatial predictors of micronutrient intake. Having ANC follow-up, family size below five, rich household wealth index, child aged 13-23 months, and children who had mothers with any job were found to have relationships with micronutrient intake among children aged 6-23 months in Ethiopia. Multicollinearity was checked by computing the Variance inflation factor (VIF). The maximum and the minimum VIF were 3.274 and 1.033, respectively. Thus, there is no significant multicollinearity between explanatory variables. Joint F-Statistic and Joint Wald Statistic result (P-value <0.001) shows that the model is statistically significant.

On the other hand, the Jarque-Bera Statistic result (P-value <0.00) explained that the OLS is biased. Furthermore, Koenker's (BP) Statistic was found to be statistically significant (P-value<0.05). Therefore, there is a possibility of heteroscedasticity and/or nonstationarity. Thus, the model is a good candidate for further regression analysis such as GWR and MGWR analysis (Table 4)**.**

Table 4: Summary of Ordinary Least Squares regression result.

Variables	Coefficients	Robust standard error	Robustt-t statistics	Robust probability	VIF
Intercept	0.442	0.059	7.522	0.000***	
Rural residence	0.068	0.039	1.737	0.083	2.984
Higher educational status of mother	0.066	0.071	0.936	0.349	1.343
Family size below five	0.054	0.044	1.228	0.0219**	1.138
Rich Household Wealth index	0.024	0.047	0.502	0.045**	3.274
Had media exposure	0.032	0.052	0.619	0.536	2.407
Had ANC follow-up	0.264	0.043	6.061	0.000***	1.406
Child age 13-23 months	0.183	0.047	3.891	0.001***	1.033
Had working/job	0.127	0.037	3.478	0.005**	1.105
	**Ordinary least square regression diagnostics**				
Number of Observations	588	AIC		124.99	
Multiple R-Squared	0.654	Adjusted R-Squared		0.612	
Joint F-Statistic	17.237	Prob(>F), (8,579) degree of freedom		0.000**	
Joint Wald Statistic	127.242	Prob(>chi-squared), (8) degree of freedom		0.000**	
Koenker's (BP) Statistic	31.96	Prob(>chi-squared), (8) degree of freedom		0.000095**	
Jarque-Bera Statistic	46.75	Prob(>chi-squared), (2) degree of freedom		0.000**	

The geographically weighted regression (GWR) analysis result

The GWR analysis showed that there was a significant improvement over global ordinary least square regression. The AICc value decreased from 124.99 for the OLS model to 107.62 for the GWR model. The difference was 17 implied that GWR best explains the spatial heterogeneity of micronutrient intake among children aged 12-23 months in Ethiopia. Besides, the adjusted R2 was 0.837, the model's ability to explain micronutrient intake has been improved by using GWR since the adjusted R2 was 0.837, indicates that GWR improved the model explaining the power of the OLS model by about 22%.

The multi-scale geographically weighted regression (MGWR) analysis result

The MGWR analysis showed that there was a significant improvement over global ordinary least square regression and GWR. The AICc value decreased from 107 for the GWR model to 102 in MGWR model. Besides, the adjusted R^2 was increased by 6 % from the GWR model. Finally, the model comparison was done by comparing AIC and R-squared values for each

model. As a result MGWR was better than OLS and GWR model since it has a highest adjusted R2 and smallest AICc value (Table 5).

Table 5: Summary of multi-scale geographically weighted regression analysis result and model comparisons

Variables	Mean	Standard deviation	Minimum	Maximum	P-value
Intercept	0.0074	0.2088	-0.5911	0.3852	
Rural residence	0.1003	0.0039	0.0937	0.1129	0.126
Higher educational status	0.0253	0.0029	0.0183	0.0342	0.266
Family size less than five	0.0478	0.1024	0.0174	0.3402	0.141
Rich Wealth index	0.0312	0.0057	0.0031	0.0484	0.026**
Had media exposure	0.0394	0.0977	0.0220	0.2785	0.060
Had ANC follow-up	0.2606	0.0662	0.1587	0.3878	0.000***
Child age 13-24 months	0.1915	0.0622	0.1207	0.3108	0.000***
Had working/job	0.1125	0.0164	0.0723	0.1393	0.000***
**Model comparison (OLS vs. GWR vs. MGWR)**					
Parameters	OLS model		GWR model	MGWR model	
AICc	124.98		107.616	102.34	
R-Squared	0.654		0.878	0.916	
Adjusted R-Squared	0.612		0.837	0.891	

In the MGWR graph revealed that a 1% increase in the child age was associated with a 19.5 % higher likelihood of taking foods rich in micronutrient, particularly in the central and southern part of Ethiopia (Addis Ababa, Gambella, and SNNPR regions). In eastern and northeastern Ethiopia (Dire Dawa, Afar, and Tigray regions), ANC follow-up was the main factor influencing the likelihood of taking foods rich in micronutrient. As the mothers had ANC follow-up, the probability of micronutrient intake rose by 26.06 %. Besides, household wealth index was the main factor influencing the likelihood of micronutrient intake. Children from rich wealth index, the probability of food intake rich in micronutrient rose by 3.1%. Additionally, with other factors held constant, children who had mothers with a job or work were 11.25 % more likely to have an adequate intake of micronutrient the northern eastern and southern parts of Ethiopia (Figure 7).

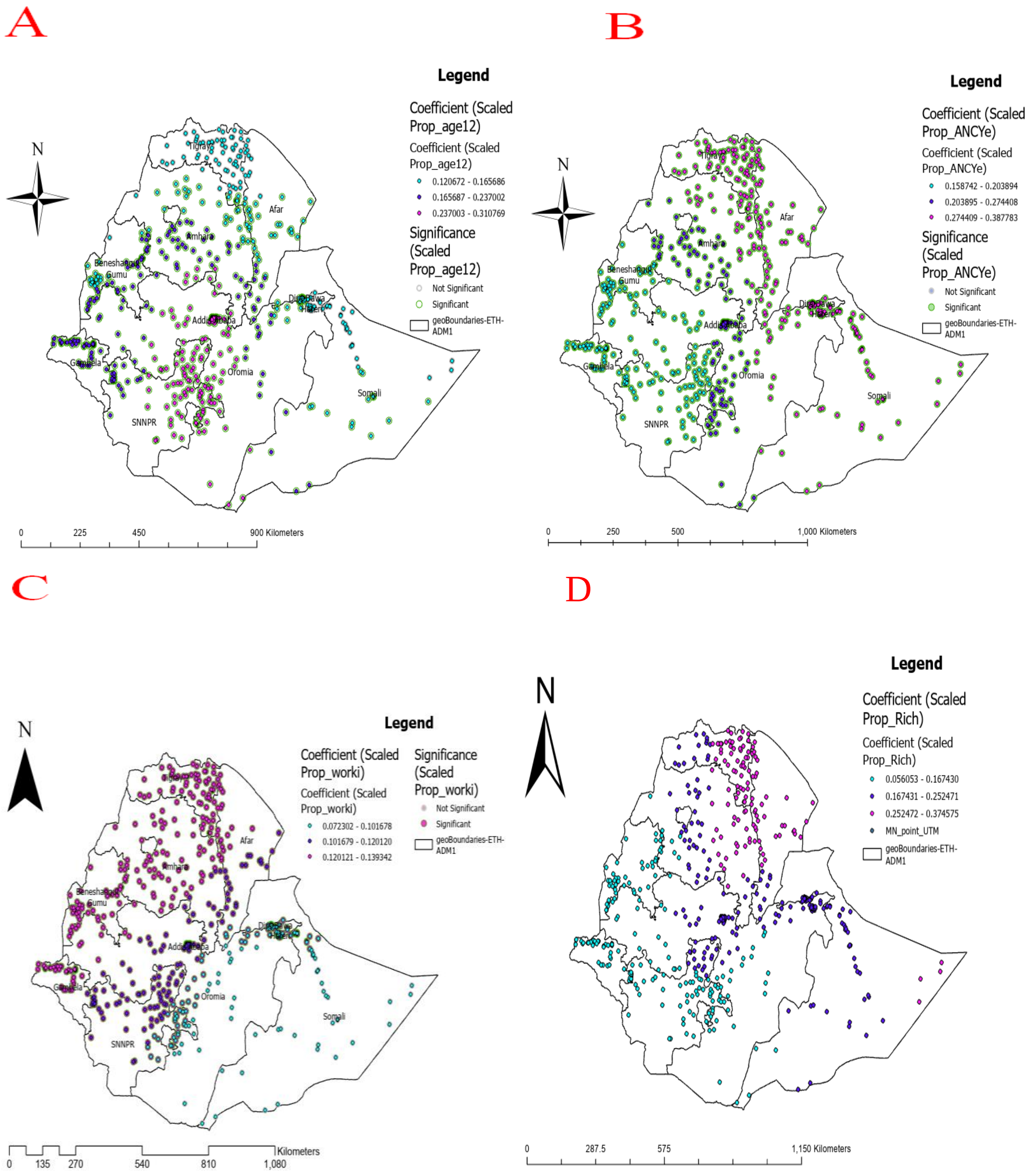

Fig 7: The spatial mapping of multi-scale geographically weighted regression coefficients by child aged 13-23 months (A), ANC follow-up (B), mother having job/work (C), and household with rich wealth index to predict the hotspot of micronutrient intake among children in Ethiopia.

Discussion

Micronutrient deficiency is a significant public health issue, especially in low and middle-income countries including Ethiopia. This study showed that only 69% (95% CI: 60.87%, 77.43%) of children aged 6-23 months had an intake of foods rich in micronutrients in Ethiopia. This result is in line with a study done in Sub-Saharan Africa (73.99%) [27] and higher than Bangladesh [4], and South Africa [28]. These discrepancies may be due to regional differences in culture, beliefs, traditions, availability and accessibility, and religious differences. The spatial distribution of micronutrient intake of children aged 6-23 months was non-random in Ethiopia. A high (Hot spots) intake status of foods rich in micronutrient were observed in Addis Ababa, Harari, Dire Dawa, Gambella, and Benishangul Gumuz regions. In line with these regions, a spatial scan statistics analysis showed that 65 primaries (most likely) clusters with the highest micronutrient intake were identified. This result is supported by the Ethiopia National Food Consumption Survey report [29]. The possible justification is that urban areas have higher income levels and better access to markets, which can result in greater availability of nutrient-rich foods, and families living in urban areas may have more purchasing power to buy foods rich in micronutrients. Moreover, urban areas may have higher levels of education and awareness regarding the importance of diversified nutrition for child development. Since Benishangul Gumuz and Gambella region are agrarian and hunter communities, they have access to serve diversified nutrients (both vegetables and animal-source foods which are rich in micronutrients) for their children.

A multi-scale geographically weighted regression analysis result showed that a family with a rich wealth index, child aged 13-23 months, mothers who have ANC follow-up, and children of mothers who have a job or work were significant factors for micronutrient intake.

Children from wealthier families have a higher probability of taking micronutrients than their counterparts. Research has consistently shown that children from wealthier households tend to have better access to a diverse and nutrient-rich diet compared to children from poorer households [2, 27, 30-32]. This is because wealthier households have greater purchasing power and resources to afford a variety of foods, including fruits, vegetables, animal-source foods, and fortified foods, which are important sources of essential vitamins and minerals. On the other hand, children from poorer households are more likely to experience food insecurity and have

limited access to nutritious foods, which can lead to deficiencies in essential micronutrients. Additionally, factors such as inadequate sanitation, poor hygiene practices, and limited access to healthcare services in low-income households can increase the inadequate intake of MN and further exacerbate the risk of MN deficiencies among children. Addressing socioeconomic inequalities in access to nutritious foods and improving the overall quality of diets among children from low-income households are important strategies for reducing micronutrient deficiencies and promoting optimal health and development. This may involve implementing targeted interventions such as food supplementation programs, nutrition education initiatives, and efforts to improve household food security and income levels. Additionally, broader policies aimed at reducing poverty, improving access to healthcare, and promoting equitable distribution of resources can also contribute to narrowing the gap in micronutrient intake between children from different socioeconomic groups [32].

The finding revealed that mothers who received ANC follow-up were associated with a 26.06% increase in the probability of their children achieving adequate micronutrient intake. The result is supported by other studies [27, 33, 34]. This is because ANC provides an opportunity for healthcare providers to assess and address health education and behavior change for mothers to be informed about optimal infant feeding practices, dietary diversity, and the importance of micronutrient-rich foods for child growth and development. The result implies that empowering mothers, who are on ANC follow-up with knowledge and skills related to nutrition, can help improve feeding practices and micronutrient intake among children. Strategies may include strengthening health systems to ensure equitable access to ANC, implementing community-based outreach programs to reach marginalized populations, integrating nutrition education into ANC curricula, and promoting collaboration between healthcare providers and community health workers, to deliver comprehensive maternal and child health services [34].

The result indicates a 19.15% increase in the probability of micronutrient intake as a child's age ranges from 13 to 23 compared to 6 to 12 month's age. This result is in congruent with many other studies conducted so far [2, 27, 33, 35]. This might be due to developmental milestones related to feeding and eating behavior, such as increased appetite, enhanced motor skills for self-feeding, and greater acceptance of a variety of solid foods. Moreover, as children become more adept at consuming a diverse range of foods, they are more likely to meet their micronutrient

requirements. Additionally, as children age from 13 to 23 months, caregivers may become more knowledgeable and skilled in providing balanced and nutritious meals, resulting in improved micronutrient intake [35]. On the other hand, children aged from 6-12 months may not start complementary foods due to behavioral and cultural practice. Public health initiatives aimed at improving infant and toddler nutrition should focus on providing education and support to caregivers, promoting breastfeeding and appropriate complementary feeding practices, and addressing barriers to accessing nutrient-rich foods. By targeting interventions during this critical period of growth and development, public health efforts can help ensure that children receive adequate nutrition for optimal health and well-being.

Children who had mothers with a job or work were 11.25% more likely to have an adequate intake of micronutrients. The result is supported by other study [2, 27, 30]. The possible justification may be due to mothers who are employed may have greater financial resources to purchase a diverse range of nutrient-rich foods for their families. Additionally, maternal employment may contribute to a more structured mealtime routine and increased access to childcare services, which can facilitate consistent and nutritious feeding practices.

Strength and limitations of the study

The utilization of nationally representative data in this study contributes to its broader applicability and statistical robustness, enabling the findings to be generalized at a national level. Furthermore, the incorporation of SaTScan and spatial distribution analysis offers valuable information regarding the geographic patterns of micronutrient intake.

However, it is important to acknowledge that the study's reliance on secondary data imposes limitations on the ability to explore the underlying causes of this phenomenon comprehensively. The data may not encompass all the factors that contribute to micronutrient intake, thereby potentially overlooking important variables. Moreover, the measurement of the consumption status of micronutrient-rich foods through a 24-hour recall approach introduces the possibility of recall and social desirability biases, which may impact the accuracy of the reported intake levels.

Conclusion and implication of the study

In Ethiopia, the intake of foods rich in MN varies across different regions. Through spatial analysis, statistically significant hot spots of high intake of these nutrient-rich foods were identified in Addis Ababa, Harari, Dire Dawa, certain parts of Benishangul, and the Gambella region.

This study revealed that the factors influencing the intake of MN-rich foods differ spatially throughout Ethiopia. Specifically, household wealth status, child aged 13-23 months, the mother's occupation, and the utilization of ANC services were found to be statistically significant predictors in various regions of Ethiopia.

These findings highlight the importance of targeted nutrition intervention programs in the identified areas to improve the intake of nutrient-rich foods among children. Encouraging ANC service utilization, providing occupation, and enhancing household wealth index are particularly recommended strategies. By addressing these contributing factors in the identified geographic locations, significant improvements in child nutrition and optimal growth can be achieved.

It should be noted that this study focused specifically on the consumption of foods rich in MN, and the association between the dependent and independent variables may be limited due to the use of secondary data. Future research should consider utilizing different datasets to assess other relevant factors associated with micronutrient intake among children, especially in areas with high levels of micronutrient insecurity.

Data availability

This study dataset used for the current study was publicly available in the DHS repository (https://dhsprogram.com).

Ethics approval and consent to participate

The 2016 EDHS was ethically reviewed by the National Research Ethics Review Committee of the Ethiopian Ministry of Science and Technology. As described in the survey final report, involvement in the survey program was voluntary, and verbal agreement (informed consent) was also taken [36]. The International Review Board of Demographic and Health Surveys program data archivists were allowed to download and use the datasets for this study. Also, the data was handled according to the Helsinki Declaration of the World Medical Association.

Consent for publication

None

Abbreviations

AICc-corrected akakie information criteria, ANC-antenatal care, GWR-geographic weighted regression, MGWR- multi-scale geographic weighted regression, MNP-multiple micronutrient powder, OLS- ordinary least square, MN-micronutrient, EDHS-Ethiopian demographic and health survey, VA-vitamin A

Funding statement

There is no specific funding for this research work.

Conflict of interest

The authors unequivocally declared that they have no conflicting interests to disclose.

Authors' contributions

ABZ: Originate the idea; formulate the protocol, acquisition of data analysis and interpretation. ABZ, AWA, TEH, and GY: Data curation, formal data analysis and manuscript write up. ABZ, RNH, and MA: Analysis, methodology, review and editing the manuscript. BDT, TAK, BBA, MAB, and RWM: data analysis and editing the manuscript. The author's consented to submit the article to the current journal.

Acknowledgement

The authors are indebted to the DHS program for giving permission to access the dataset.

References

1. Harika, R., et al., Micronutrient status and dietary intake of iron, vitamin A, iodine, folate and zinc in women of reproductive age and pregnant women in Ethiopia, Kenya, Nigeria and South Africa: a systematic review of data from 2005 to 2015. Nutrients, 2017. **9**(10): p. 1096.
2. Gebeye, L.G., E.Y. Dessie, and J.A. Yimam, Predictors of micronutrient deficiency among children aged 6–23 months in Ethiopia: a machine learning approach. Frontiers in Nutrition, 2024. **10**: p. 1277048.
3. The Federal Democratric Republic of Ethiopia, Government of Ethiopia National Nutrition Program, 2016-2020. July 2016.

4. Ahmed, F., N. Prendiville, and A. Narayan, Micronutrient deficiencies among children and women in Bangladesh: progress and challenges. Journal of nutritional science, 2016. **5**: p. e46.
5. UNICEF, The State Of the World's Children 2019: Children, food and nutrition: Growing well in a changing world. 2019.
6. Organization(WHO), W.H., Multiple micronutrient powders for point-of-use fortification of foods consumed by children 2–12 years of age. Aug,2023.
7. Walker, S.P., et al., Child development: risk factors for adverse outcomes in developing countries. The lancet, 2007. **369**(9556): p. 145-157.
8. Gebremedhin, T., et al., Micronutrient intake status and associated factors among children aged 6–23 months in the emerging regions of Ethiopia: A multilevel analysis of the 2016 Ethiopia demographic and health survey. PloS one, 2021. **16**(10): p. e0258954.
9. Government of Ethiopia, U.C.s.F., UNICEF, National Food and Nutrition Strategy Baseline Survey - Key Findings Preliminary Report. 23 march 2023.
10. Belay, D.G., et al., Individual and community level determinants of iron intake among children 6-59 months old in Ethiopia: multilevel logistic regression analysis. BMC Pediatr, 2022. **22**(1): p. 661.
11. USAID, Ethiopia: Nutrition Profile. 2021.
12. Bhandari, N., R. Bahl, and S. Taneja, Effect of micronutrient supplementation on linear growth of children. British Journal of Nutrition, 2001. **85**(S2): p. S131-S137.
13. Health, E.F.M.o., GUIDELINES FOR THE PREVENTION AND CONTROL OF MICRONUTRIENT DEFICIENCIES 2016.
14. Kawai, K., et al., Maternal multiple micronutrient supplementation and pregnancy outcomes in developing countries: meta-analysis and meta-regression. Bulletin of the World Health Organization, 2011. **89**: p. 402-411.
15. Engidaw, M.T., et al., Micronutrient intake status and associated factors in children aged 6-23 months in sub-Saharan Africa. Sci Rep, 2023. **13**(1): p. 10179.
16. Demographic, I., Health Survey Sampling and Household Listing Manual. Calverton: Measure DHS, 2012.
17. Croft, T.N., et al., Guide to DHS statistics. Rockville: ICF, 2018. **645**.

18. Fotheringham, A.S., C. Brunsdon, and M. Charlton, Geographically weighted regression: the analysis of spatially varying relationships. 2003: John Wiley & Sons.
19. Waldhör, T., Spatial Autocorrelation Coefficient, Moran's. Wiley StatsRef: Statistics Reference Online, 2014.
20. Krivoruchko, K., Empirical bayesian kriging. ArcUser Fall, 2012. **6**(10): p. 1145.
21. Kulldorff, M., A spatial scan statistic. Communications in Statistics-Theory and methods, 1997. **26**(6): p. 1481-1496.
22. Haque, U., et al., Modelling malaria treatment practices in Bangladesh using spatial statistics. Malaria journal, 2012. **11**(1): p. 1-11.
23. Comber, A., et al., The GWR route map: a guide to the informed application of Geographically Weighted Regression. arXiv preprint arXiv:2004.06070, 2020.
24. Li, Z. and A.S. Fotheringham, Computational improvements to multi-scale geographically weighted regression. International Journal of Geographical Information Science, 2020. **34**(7): p. 1378-1397.
25. Elsayed, A.M.M., Using Different Methods to Overcome Modeling Problems.
26. Nazeer, M. and M. Bilal, Evaluation of ordinary least square (OLS) and geographically weighted regression (GWR) for water quality monitoring: A case study for the estimation of salinity. Journal of Ocean University of China, 2018. **17**: p. 305-310.
27. Engidaw, M.T., et al., Micronutrient intake status and associated factors in children aged 6–23 months in sub-Saharan Africa. Scientific Reports, 2023. **13**(1): p. 10179.
28. Faber, V.J., AJS Benadé, Mieke, Nutritional status and dietary intakes of children aged 2–5 years and their caregivers in a rural South African community. International journal of food sciences and nutrition, 2001. **52**(5): p. 401-411.
29. Gebru, M., et al., Ethiopia’s food treasures: revitalizing Ethiopia’s underutilized fruits and vegetables for inclusion in the Food-Based Dietary Guidelines for improved diet diversity, nutrition and health of the population. 2019.
30. Tiruneh, S.A., et al., Spatial distribution and geographical heterogeneity factors associated with poor consumption of foods rich in vitamin A among children age 6–23 months in Ethiopia: Geographical weighted regression analysis. PloS one, 2021. **16**(6): p. e0252639.

31. Tiruneh, S.A., et al., Spatial distribution of iron rich foods consumption and its associated factors among children aged 6–23 months in Ethiopia: spatial and multilevel analysis of 2016 Ethiopian demographic and health survey. Nutrition Journal, 2020. **19**: p. 1-13.
32. Saaka, M. and S.M. Osman, Does household food insecurity affect the nutritional status of preschool children aged 6-36 months? International Journal of Population Research, 2013. **2013**.
33. Akalu, Y., et al., Iron-rich food consumption and associated factors among children aged 6–23 months in sub-Saharan Africa: A multilevel analysis of Demographic and Health Surveys. Plos one, 2021. **16**(6): p. e0253221.
34. Semagn, B.E., Z.A. Gebreegziabher, and W.A. Abebaw, Iron-rich food consumption and associated factors among children aged 6–23 months in Sierra Leone: multi-level logistic regression analysis. BMC Public Health, 2023. **23**(1): p. 1793.
35. Gewa, C.A. and T.F. Leslie, Distribution and determinants of young child feeding practices in the East African region: demographic health survey data analysis from 2008-2011. Journal of Health, Population and Nutrition, 2015. **34**: p. 1-14.
36. Ethiopian Public Health Institute (EPHI) and ICF, Ethiopia Mini Demographic and Health Survey. Final Report. Rockville, Maryland, USA: EPHI and ICF. 2019.
