# Supplementary material for: Spatial distribution and determinants of micronutrient intake status among children aged 6–23 months in Ethiopia: A Multi-scale Geographical Weighted Regression Analysis
Source: PLoS One. 2025 Apr 29;20(4):e0321113. doi: 10.1371/journal.pone.0321113 (PMC12040264; doi:10.1371/journal.pone.0321113)
Supplement: Attachment — Submitted filename: comments.docx [file pone.0321113.s001.docx]

1.      Need to provide more information about the location (coordinates) geo-scrambling/dithering; why it’s necessary, how it affects your analysis and how you mitigated for it in the analysis


2.     You need a summary section of all the methods which may be interconnected with a clear simple figure, to show what feeds where and why all the methods are necessary. It seems hotspot analysis, SaTScan analysis and spatial prediction(kriging) all point towards the same message. Therefore this summary paragraph early on will be useful for the readers.


3.     Also, note that the values of zero indicate perfect randomness.

4.     Likewise, provide a preamble to local Getis Ord statistics for the reader

5.     On Spatial interpolation, need to use a variogram to test the spatial dependence for micronutrients intake including details of practical range.

6.     What covariates were used for spatial prediction of short birth interval? These need to be specified and included. Also, put down the equation for easier comprehension. State-of-the-art model-based geostatistics would be a better fit to fully exploit the posterior samples than kriging. Therefore justify your choice of the modelling approach

7.     Multilevel modelling should maybe come in before the spatial prediction so that the covariates are included during spatial prediction for robust results

8.     On Statistical analysis, the statement “Finally, the mixed effect model, which included both fixed and random effect variables were fitted. To include the variable in the model p-value.”; seems to be incomplete and in the wrong place

9.     On SaTScan analysis, please state the unit of analysis. Does the SaTScan analysis and hotspot analysis provide similar insights? Why was it important to have these two in the paper?

10.     On Multilevel binary logistic analysis, why was clustering considered only at the cluster level? Need to account for the fact that individuals are within households, households within clusters and clusters within a region

11     The discussion has a big section which reads like a repetition of the results section. Please edit this section to include details on what this study means to Ethiopia and the global community in terms of policy and actions that can be taken

12.     Given the importance of subnational targeting, why were districts and/or region-level estimates not presented? These would align with units of decision-making and can be obtained by aggregating results of spatial prediction. The single national value masks heterogeneity while the pixel map is not used as a policy actionable unit

13.      Under limitations- include geo-scrambling of coordinates if not accounted for in the analysis

14. The document needs language edition

15. You have published with other method of analysis? So would you think that it will provides another insights? I need a clear answer. Unless biuased.
