# Supplementary material for: Spatial distribution and determinants of micronutrient intake status among children aged 6–23 months in Ethiopia: A Multi-scale Geographical Weighted Regression Analysis
Source: PLoS One. 2025 Apr 29;20(4):e0321113. doi: 10.1371/journal.pone.0321113 (PMC12040264; doi:10.1371/journal.pone.0321113)
Supplement: Attachment — Submitted filename: Response to reviewer.docx [file pone.0321113.s003.docx]

**January 31/2025**

**Point by point response letter**

**Subject: submission of revised manuscript and point by point response for reviewers’ comments**

**Manuscript ID**: PONE-D-24-22661

**Manuscript title**: Spatial distribution and determinants of micronutrient intake status among children aged 6-23 months in Ethiopia: A Multi-scale Geographical Weighted Regression Analysis

**To: - Plos one**

**Dear respected editors and reviewers,**

We would like to thank you for these constructive, building, and improvable comments on this
manuscript that would improve the substance and content of the manuscript. We have considered each comments and clarification questions of reviewers on the manuscript thoroughly. Our
point-by-point responses for each comment and question are described in detail on the following
pages. Furthermore, the details of changes were shown by track changes using red color on the recently attached file labeled “revised manuscript with track changes” along with this we have also uploaded the detail point by point response labeled “ response to reviewers”. The manuscript language was checked by language professionals and we have followed the journal guidelines upon preparation and submission of the manuscript.

Dear Dr. Zemariam,

Thank you for submitting your manuscript to PLOS ONE. After careful consideration, we feel that it has merit but does not fully meet PLOS ONE’s publication criteria as it currently stands. Therefore, we invite you to submit a revised version of the manuscript that addresses the points raised during the review process. We look forward to receiving your revised manuscript.

Kind regards,

Samuel Kofi Tchum, Ph.D.

Academic Editor

PLOS ONE

**Authors’ response**: Dear esteemed Editor, thank you for managing our manuscript and offering detailed feedback. We have provided justifications and revised the manuscript accordingly. Please find the revised manuscript attached, along with our point-by-point responses.

Journal Requirements:

When submitting your revision, we need you to address these additional requirements.

1. Please ensure that your manuscript meets PLOS ONE's style requirements, including those for file naming.

2. Your ethics statement should only appear in the Methods section of your manuscript. If your ethics statement is written in any section besides the Methods, please move it to the Methods section and delete it from any other section. Please ensure that your ethics statement is included in your manuscript, as the ethics statement entered into the online submission form will not be published alongside your manuscript.

**Authors’ response**: Dear, we thank you and appreciate your suggestions. We have carefully considered the journal guideline up on submission of the revised manuscript and moved the ethical statement to the methods section. Kindly see the revision on the updated manuscript.

3. We note that [Figures 1,4,5,6 and 7] in your submission contain [map/satellite] images which may be copyrighted. We require you to either (1) present written permission from the copyright holder to publish these figures specifically under the CC BY 4.0 license, or (2) remove the figures from your submission:

a. You may seek permission from the original copyright holder of Figures 1,4,5,6 and 7 to publish the content specifically under the CC BY 4.0 license.

b. If you are unable to obtain permission from the original copyright holder to publish these figures under the CC BY 4.0 license or if the copyright holder’s requirements are incompatible with the CC BY 4.0 license, please either i) remove the figure or ii) supply a replacement figure that complies with the CC BY 4.0 license. If applicable, please specify in the figure caption text when a figure is similar but not identical to the original image and is therefore for illustrative purposes only. Please respond to the following queries regarding Figure 1, 4, 5, 6, and 7:
-Where did the authors obtain the maps, basemaps, shapefiles, map data, etc in Figures 1, 4, 5, 6, and 7?

**Authors’ response**: Thank you for your feedback regarding Figures 1, 4, 5, 6, and 7. We generated these maps using ArcGIS Pro software and assure you that all figures are original, based on the GPS data we accessed from the following websites. We mistakenly attached the incorrect link and apologize for the inconsistency. We used the Africa Geo Portal for the previously published article entitled “Spatial Variation, 20-Year Trends, and Determinants of the Double Burden of Wasting and Stunting Among Under-Five Children in Ethiopia: A Geo-Spatial and Multivariate Decomposition Analysis (2000–2019) and Mapping Fertility Rates at National, Sub-National, and Local Levels in Ethiopia Between 2000 and 2019,” but it was not used in this study. Therefore, all of the figures listed above contain map data retrieved from the DHS Program through the link below.

[**https://spatialdata.dhsprogram.com/data/#/**](https://spatialdata.dhsprogram.com/data/#/)

The reviewers' comments are as follows;

**Reviewer#1**

1. Need to provide more information about the location (coordinates) geo-scrambling/dithering; why it’s necessary, how it affects your analysis and how you mitigated for it in the analysis

**Authors’ response**: Dear esteemed reviewer, thank you for your insightful feedback. We appreciate your guidance in enhancing the clarity and rigor of our manuscript. We sincerely apologize for missing such an important analysis. The authors initially did not consider this analysis for the following several reasons. However, we now understand the relevance of geo-scrambling and have acknowledged it as a limitation, suggesting it for future researchers. Kindly see the revised manuscript.

 **Aggregate Data Usage**: The EDHS data is often analyzed at the aggregate level, where the focus is on broader trends rather than individual coordinates, making geo-scrambling unnecessary.

 **Established Protocols**: The EDHS employs standardized methodologies that do not typically incorporate geo-scrambling, ensuring consistency with previous studies and facilitating comparability.

 **Focus on Key Variables**: The analysis emphasizes key demographic and health variables that are more pertinent to the research questions, reducing the relevance of geo-scrambling.

 **Data Integrity and Quality**: The EDHS data is collected using rigorous methods, ensuring that the geographic information is reliable and valid without the need for scrambling.

 **Privacy Considerations**: The analysis already adheres to ethical standards for data privacy, and geo-scrambling may not add significant value given existing privacy safeguards.

 **Contextual Relevance**: Geographic context and regional patterns are crucial in the analysis, and geo-scrambling could obscure important insights about health disparities and access to services.

2. You need a summary section of all the methods which may be interconnected with a clear simple figure, to show what feeds where and why all the methods are necessary. It seems hotspot analysis, SaTScan analysis and spatial prediction (kriging) all point towards the same message. Therefore this summary paragraph early on will be useful for the readers.

**Authors’ response**: Dear esteemed reviewer, thank you for your thoughtful comments and suggestions. In response, we have incorporated the rationale and assumptions behind the analyses mentioned, as well as the reasoning for conducting additional analyses such as OLS, GWR, and MGWR. This should help readers better understand the basis for each method and how they collectively reinforce our conclusions. We truly appreciate your valuable feedback.

3. Also, note that the values of zero indicate perfect randomness. Likewise, provide a preamble to local Getis Ord statistics for the reader

**Authors’ response**: Dear esteemed reviewer, thank you for your constructive feedback. In response, we have added a clear explanation in the revised manuscript to clarify this point for readers. Additionally, we have included a preamble to the local Getis-Ord statistics, providing essential context and background information. This should enhance the reader's understanding of the methodology and its significance in our analysis. Thank you once again for your valuable insights.

4. On Spatial interpolation, need to use a variogram to test the spatial dependence for micronutrients intake including details of practical range.

**Authors’ response**: Dear esteemed reviewer, thank you for your insightful comment. While variogram analysis is a powerful tool in geostatistics for quantifying spatial variability, there are several situations where it may not be appropriate or necessary. Here are some scenarios where variogram analysis cannot be conducted:

- Insufficient Data: If the dataset is too small, the variogram may not provide reliable estimates of spatial dependence.
- Sparse Data Distribution: When data points are very sparse or unevenly distributed, it can lead to unreliable variogram calculations.
- Irregular Sampling Patterns: If the sampling locations are too irregular or clustered, the variogram may not effectively represent the spatial structure.
- High Variability or Noise: If the data is extremely noisy, variogram analysis may be misled by measurement error or random variability rather than true spatial patterns.
- Non-Stationarity: In cases where the mean or variance of the data changes over space, traditional variogram analysis (which assumes stationarity) may not be suitable.
- Categorical Data: For categorical or binary data, variogram analysis may not be appropriate. Instead, techniques like indicator kriging or other methods for categorical data should be used.
- Alternative Methods Available: In some cases, simpler methods (e.g., regression analysis, Kriging, IDW) may provide sufficient insight without the complexity of variogram analysis.

Generally, while variogram analysis is a valuable tool in many contexts, its applicability depends on the nature of the data and the specific research questions. Understanding when not to use it can help practitioners choose more suitable methods for analyzing spatial variability.

5. What covariates were used for spatial prediction of short birth interval? These need to be specified and included. Also, put down the equation for easier comprehension. State-of-the-art model-based geostatistics would be a better fit to fully exploit the posterior samples than kriging. Therefore justify your choice of the modelling approach.

**Authors’ response**: Dear esteemed reviewer, we have included the covariated used for prediction of short birth interval. In addition, while state-of-the-art model-based geostatistics offers advanced capabilities, kriging was appropriate given the nature of our data and the need for interpretable results. Here are some scenarios where using kriging is used:

- Simplicity and Interpretability: Kriging is easier to understand and implement, making it suitable for practitioners who may not have extensive statistical expertise.

- Clear Output: The results from kriging are often more intuitive and easier to explain to non-specialist stakeholders.

- Small to Moderate Datasets: In cases with small or moderate datasets, kriging can provide reliable estimates without the need for more complex modeling approaches.
- Assumption of Stationarity: If the spatial process is known or assumed to be stationary (constant mean and variance), kriging is highly effective, as it relies on this assumption.
- Computational Efficiency: For smaller datasets, kriging is computationally less intensive than some advanced model-based approaches, making it quicker for generating predictions.
- Focus on Local Predictions: Kriging provides localized predictions and uncertainty estimates, which can be particularly useful in applications where local variation is critical (e.g., environmental monitoring).
- Standard Practice in Certain Fields: Established Methodology: In some disciplines (like health, mining or environmental science), kriging is a well-accepted standard, and its use may be expected or preferred by the community.
- Interpretation of Variance-Uncertainty Quantification: Kriging explicitly provides estimates of prediction uncertainty, which can be crucial for decision-making in resource management or environmental assessments.

**Generally**, while state-of-the-art model-based geostatistics offers advanced capabilities, kriging remains a valuable tool in many contexts, particularly when simplicity, computational efficiency, and a strong understanding of spatial data structure are paramount.

6. Multilevel modeling should maybe come in before the spatial prediction so that the covariates are included during spatial prediction for robust results

**Authors’ response**: Dear esteemed reviewer, thank you for your constructive feedback. We appreciate your suggestion that multilevel modeling should come before spatial predictions to better integrate covariates and strengthen our results. While we see the advantages of this approach, we have only treated region as a community-level variable and noting this in the limitations section of the manuscript. Your insightful comments have greatly enhanced our understanding of the methodology. Thank you once again for your valuable input.

7. On Statistical analysis, the statement “Finally, the mixed effect model, which included both fixed and random effect variables were fitted. To include the variable in the model p-value.”; seems to be incomplete and in the wrong place.

**Authors’ response**: Dear reviewer, we appreciate your observation regarding the statement on statistical analysis. We have revised this section to ensure clarity and proper placement, providing a more coherent explanation of our statistical methodology. Thank you for pointing this out.

8. On SaTScan analysis, please state the unit of analysis. Does the SaTScan analysis and hotspot analysis provide similar insights? Why was it important to have these two in the paper?

**Authors’ response**: Dear esteemed reviewer, we thank you for your insightful question regarding the SaTScan analysis.

- **Unit of Analysis**: In our study, the unit of analysis for the SaTScan analysis was defined as geographic regions, specifically using counties. This allowed us to effectively assess the spatial distribution of events over time.
- **Comparison of Insights**: While both SaTScan analysis and hotspot analysis aim to identify high-incidence areas, they offer different perspectives. SaTScan focuses on statistically significant clusters, accounting for both spatial and temporal data, which helps in identifying areas with a higher-than-expected number of events. On the other hand, hotspot analysis identifies areas with high concentrations of events, which may not necessarily be statistically significant. Thus, while there may be overlap in the identified areas, the insights derived from each method can differ.
- **Importance of Both Analyses**: Incorporating both SaTScan and hotspot analysis in our paper strengthens our findings. SaTScan's rigorous statistical approach lends credibility to our identification of significant clusters, while hotspot analysis highlights areas that may warrant additional attention or intervention. Together, these methods provide a more comprehensive understanding of the spatial dynamics in our data, ultimately supporting more informed public health decision-making.

We appreciate your feedback and hope this response clarifies the rationale behind our analytical choices.

9. On Multilevel binary logistic analysis, why was clustering considered only at the cluster level? Need to account for the fact that individuals are within households, households within clusters and clusters within a region

**Authors’ response**: Dear respected reviewer, we thank you for your valuable feedback regarding the multilevel binary logistic analysis. We acknowledge the importance of accounting for the hierarchical structure of the data, specifically the nesting of individuals within households, households within clusters, and clusters within regions. Our decision to focus clustering only at the cluster level was primarily based on the initial analysis of the data structure and the computational feasibility of the model given the sample size and complexity.

However, we recognize that a more granular approach, which considers the nested nature of the data, could provide a more accurate estimation of the standard errors and enhance the robustness of our findings. In this study, we have to put it as a limitation of the study and suggest for future researchers to incorporate a multilevel model that accounts for the full hierarchy, allowing us to better capture the variance at each level (individual, household, and cluster).

We appreciate your suggestion and will consider this in our ongoing work to ensure that our analyses reflect the true structure of the data and provide more reliable conclusions. Thank you for helping us improves the quality of our research.

10. The discussion has a big section which reads like a repetition of the results section. Please edit this section to include details on what this study means to Ethiopia and the global community in terms of policy and actions that can be taken

**Authors’ response:** Dear esteemed reviewer, thank you for your insightful comment on the discussion section. We will revise this part to reduce overlap with the results and focus on the implications of our findings:

1. **Contextual Relevance**: We have clarified how our results address public health challenges in Ethiopia.
2. **Policy Implications**: Actionable recommendations for policymakers have been included, such as targeted interventions and resource allocation strategies.
3. **Global Perspective**: We have highlighted the study's contribution to global health discussions, emphasizing collaborative efforts in addressing shared challenges.
4. **Call to Action**: Specific actions for stakeholders, including government and NGOs, have been suggested to enhance coordinated responses to health issues.

These revisions will strengthen the discussion and underscore the significance of our findings for both Ethiopia and the global community. Thank you once again for your concrete comments.

11. Given the importance of subnational targeting, why were districts and/or region-level estimates not presented? These would align with units of decision-making and can be obtained by aggregating results of spatial prediction. The single national value masks heterogeneity while the pixel map is not used as a policy actionable unit

**Authors’ response:** Dear reviewer, thank you for your thoughtful comment. In our initial analysis, we opted to present a single national value to provide a broad overview of the findings for a few key reasons:

- **Simplicity and Clarity**: A national estimate provides a straightforward summary for readers, allowing for easier comprehension of the overall trends without overwhelming them with data.
- **Initial Focus**: Our primary aim was to highlight the overall magnitude of the issue at a national level, which can sometimes be more impactful for broad policy discussions.
- **Comparative Context**: Presenting a national value allows for easier comparison with other national studies or benchmarks, facilitating a broader understanding of the issue within a global context.

However, we recognize the importance of subnational targeting and will incorporate district and region-level estimates in our revisions to enhance the actionable insights for policymakers. Thank you for bringing this to our attention.

12. Under limitations- include geo-scrambling of coordinates if not accounted for in the analysis. The document needs language edition

**Authors’ response**: Thank you for your valuable feedback. We have included a specific mention of geo-scrambling of coordinates in the limitations section if it was not adequately accounted for in our analysis. This is an important consideration, as it can impact the accuracy of spatial predictions and the interpretations of our findings. We have added it in the revised version.

Additionally, we have conducted a thorough language review of the document to enhance clarity and readability. We appreciate your suggestions, as they will help improve the overall quality of our manuscript. Thank you for your guidance.

13. You have published with other method of analysis? So would you think that it will provides another insights? I need a clear answer. Unless biased.

**Authors’ response**: Dear esteemed reviewer, we thank you for your inquiry regarding the potential of multiscale geographic weighted regression (MGWR) in our analysis.

**Yes**, we have experience with machine learning methods in our previous publications. We believe that applying MGWR could indeed provide valuable insights, as it accounts for spatial heterogeneity and allows for varying relationships across different scales. This approach can help identify localized effects that may be missed with traditional regression models.

In summary, we see significant potential in using MGWR to enhance our understanding of the data and to inform policy decisions more effectively. Thank you for your insightful question.

**Reviewer#2**

**Introduction**

The introduction effectively highlights the global and national impact of micronutrient (MN) deficiency, particularly among children in Ethiopia. However, it could be improved by refining its structure, enhancing clarity, and avoiding repetition. Here are some suggestions:
1. It would be better to add a specialized definition of malnutrition at the beginning of the introduction.

**Authors’ response**: Dear esteemed reviewer, we thank you for your suggestion. We agree that providing a clear and concise definition will enhance the reader's understanding of the term and its relevance to our study. In the revised manuscript, we will incorporate a specialized definition of malnutrition, along with its various forms, to set a solid foundation for our discussion. We appreciate your constructive feedback, which will help improve the clarity of our introduction. Thank you for your guidance to improve our manuscript.

2. For improving the coherence and clarity of the introduction section, you can proceed based on the following breakdown and provide an explanation.

a. In the first paragraph, you can briefly explain the following topics mentioned in the introduction: MN deficiency as a global public health concern, the importance of the first 1,000 days of a child's life, global scale of malnutrition, and vulnerability in Low- and middle-income countries.
b. In the second paragraph, you can discuss the importance of adequate micronutrient intake, provide definitions and classifications of micronutrients, and address other related issues concerning the mentioned micronutrients.

c. In the third paragraph, discuss strategies related to reducing malnutrition, the recommendations from the World Health Organization, the effectiveness of supplementation programs, and factors influencing malnutrition.

d. Finally, identify the existing gaps, and state your objective

**Authors’ response**: Dear respected reviewer, we appreciate your vital suggestions. We have accepted your recommendation and revised the manuscript accordingly.

**Method**
The methods section is thorough and detailed but could benefit from improved organization. Here are some suggestions:

1. Divide the methods section into subsections such as "Data Source," "Study Population," "Variables and Measurements," "Data Processing and Analysis," and "Model Evaluation" for better readability. Use a reporting guideline suited to your article to better organize headings and enhance clarity and order.

**Authors’ response**: Dear esteemed reviewer, we thank you for your suggestion to restructure the methods section. We agree that dividing it into subsections will improve readability and clarity. We have implemented this organization in the revised manuscript, using an appropriate reporting guideline to enhance the overall structure. Your feedback is greatly appreciated.

2. Depending on the authors' preferences, explain why specific techniques, like Multi-Scale Geographically Weighted Regression (MGWR), were chosen over others, such as Geographically Weighted Regression (GWR). Providing a rationale for the superiority of MGWR could help clarify its advantages.

**Authors’ response**: Dear reviewer, we thank you for raising such an important comments. Authors choose MGWR over GWR due to several key reasons:

- **Handling Spatial Scale Variability**
- **MGWR** allows for the analysis of spatial relationships at multiple scales, recognizing that different geographic areas may have different underlying processes. This flexibility enables researchers to capture complex spatial dynamics that GWR may miss, as GWR typically assumes a single scale of influence for the entire study area.
- **Improved Model Fit**
- By incorporating multiple scales, MGWR can provide a better fit to the data. It allows for localized parameter estimates that can vary not only by location but also by the scale of the relationships being modeled. This can lead to more accurate predictions and a clearer understanding of the spatial variability in the data.
- **Enhanced Interpretability**
- MGWR offers more nuanced insights into spatial relationships. By examining how relationships change across different scales, researchers can identify specific factors that may be more relevant in certain areas. This level of detail can improve policy-making and targeted interventions.
- **Reduction of Multicollinearity**
- MGWR can mitigate issues of multicollinearity that may arise in traditional GWR when predictors are correlated. By accommodating varying relationships at different scales, MGWR can help to isolate the effects of individual predictors more effectively.
- **Robustness to Spatial Autocorrelation**
- The ability of MGWR to account for spatial autocorrelation at various scales makes it a more robust choice in many cases. This is particularly important in spatial data analysis, where ignoring autocorrelation can lead to biased results.
- **Advanced Computational Techniques**
- MGWR utilizes modern computational techniques to estimate parameters more efficiently and effectively. This allows researchers to handle larger datasets and more complex models without significant increases in computation time.

In summary, the choice of MGWR over GWR is often driven by the need for greater flexibility, improved model performance, enhanced interpretability, and a more robust handling of spatial relationships. These advantages make MGWR a powerful tool in spatial analysis, particularly in contexts where understanding the variability of relationships across different scales is crucial.

**Results**
The results section offers a detailed and data-rich summary but could be improved for clarity and precision:
1. Amend the grammatical error in the first line from "More than one-third (34.5%) of the female children were had taken foods rich in micronutrient" to "More than one-third (34.5%) of the female children had consumed foods rich in micronutrients."

It might be beneficial to summarize key findings more clearly. For instance, explicitly state the regions with the highest and lowest micronutrient intake before discussing the methods used.

**Authors’ response**: We thank you for your valuable feedback. We have accepted your vital suggestion and made amendments in the revised manuscript. We appreciate your suggestions, which have helped improve the clarity and readability of our work. Thank you for your insightful comments.

**Discussion**
The discussion is generally well-structured and comprehensive but could be enhanced by improving clarity, conciseness, and emphasis:

1. Shorten long sentences for better clarity. For example, sentence 33 starting with "Addressing socioeconomic inequalities..." could be split into two sentences.

Avoid repeating explanations about previously discussed points, such as the role of wealth in influencing micronutrient intake. Consolidate these discussions for brevity.

**Authors’ response**: Dear reviewer, we thank you for your important suggestion. We have accepted it and made a revision accordingly.

**Limitations**
1. While limitations are acknowledged, they could be expanded to address potential confounders or biases not controlled for in the study. Discuss factors like seasonal variations in food availability or regional economic disparities as additional limitations.

**Authors’ response**: Dear esteemed reviewer, we thank you for your vital recommendation to enhance the quality of the manuscript. We have accepted your suggestion and revised it accordingly. Kindly review the revised manuscript.

**Reviewer#3**

The manuscript details an interesting piece of research, and seems technically sound. "Hidden hunger" is a key public and global health concern, especially in infants in lower-income countries, and in light of climate change pressurizing the situation. The statistical analysis is complex and thorough, and appropriate to answer the research questions. The conclusions are the same key messages that the reader draws from the data. There are a few clarifications that I would like the authors to make, as well as some grammatical areas I have highlighted and commented on the attachment. All of my comments can be read on the PDF. Overall, a very good study and clearly written paper.

1. Used for binary models - check this in Methods section.

**Authors’ response**: Dear esteemed reviewer, we thank you for the issue and we have revised the manuscript. The Bernoulli model is a binary model. It is used to model situations where there are two possible outcomes. The application of this model enabled us to identify significant clusters in the data, providing insights into the spatial distribution of the outcomes.

2. Around 38% of children aged 6-59 months in urban areas and 24% in rural areas suffer from either iron or folate deficiency, with iron deficiency being a primary cause of anemia, affecting 54% of children in this age group according to 2016 Ethiopian demographic and health survey (EDHS) report only 9.24% of children take iron [9, 10]. Additionally, vitamin A deficiency alone is responsible for 80,000 deaths among preschool children in Ethiopia, with only 63% of children nationwide receiving adequate supplementation which is far from the government plan which is 90%.

-This needs to be broken down into a couple of sentences as very confusing.

-Could change last 'which is' to 'of' in order to make the sentence structure work here.

**Authors’ response**: Dear esteemed reviewer, we thank you for your vital suggestion. We have accepted and revised it accordingly. Thank you once again for your fruitful suggestions.

3. ANC follow up of the mother, working status,

-What is ANC follow-up?

-Of both parents? As parental/maternal has been specified here before?

-What do you mean by geography and racial differences?

**Authors’ response**: Dear reviewer, we thank you for the comment. ANC stands for Antenatal Care. ANC follow-up refers to the regular medical check-ups that a pregnant woman receives throughout her pregnancy. These visits are crucial for monitoring the health of both the mother and the developing fetus, providing necessary interventions, and educating mothers about prenatal care. By geography, we refer to the variations in health outcomes and access to healthcare services based on different regions within Ethiopia. This can include urban versus rural settings and disparities that exist in different provinces. Racial differences pertain to the variations in health outcomes observed among different ethnic groups within the country, which can be influenced by cultural, socioeconomic, and environmental factors. I have revised it. Kindly see the revision made in the updated manuscript.

4. Think this could be made slightly clearer, I'm not quite sure of the difference here between what was the target population and what actually were the study pop?

**Authors’ response**: Dear respected reviewer, thank you for your insightful question.

The **target population** for this study includes all living children aged 6-23 months along with their mothers throughout Ethiopia. This represents the broader group we aim to understand and draw conclusions about.

In contrast, the **study population** specifically refers to the children aged 6-23 months who were located in the selected enumeration area from which we draw a data. This is a subset of the target population, reflecting the practical limitations of our study in terms of location and accessibility. We hope this distinction clarifies our methodology and the scope of our research. Thank you for your attention to this detail.

5. With the measurement of MN rich food 'within last 24 hours'. Seems cross-sectional, so might be subject to seasonal factors where foods are plentiful (if that's when the data collection happened), or e.g. the day after market day when the households have more good quality food. Or any other factor that might introduce bias in a 24-hour window - included in limitations?

**Authors’ response**: Dear esteemed reviewer, thank you for your insightful comment on measuring micronutrient-rich foods within the last 24 hours. We acknowledge that our cross-sectional design may introduce biases related to seasonal variations and timing, such as proximity to market days. We have included these limitations in the revised manuscript and emphasize the need for caution in interpreting our findings. Thank you for highlighting this important issue.

6. Food groups or more generally supplementation/intervention? Be best to clarify here as seems to be follow-on from VAS and deworming treatment. This has previously been shortened to an abbreviation (MN) so keep this consistent.

**Authors’ response**: Dear esteemed reviewer we thank you for your suggestion. We have accepted and corrected it accordingly. Kindly see the revised manuscript.

7. Desire more children classification as no more want, undecided and wants more are unclear here.

**Authors’ response**: Dear esteemed reviewer we thank you for raising such an important concern. To clarify the classification regarding desires for more children, we could break it down into three distinct categories:

1. **No More Children**: Individuals who have decided they do not want any more children. This may include those who feel content with their current family size or have decided against expanding their family due to personal, financial, or health reasons.
2. **Undecided**: Individuals who are uncertain about their desire for more children. This group may be weighing various factors, such as their current life situation, financial stability, or personal aspirations, and have not reached a definitive decision.
3. **Wants More Children**: Individuals who express a clear desire to have more children. This group may actively be planning for future children or are open to the possibility of expanding their family in the future.
8. Sex of Household Head? Of participant?

Had cough----Is this related to the child or the mother? Also is there a timeframe for this? .i.e. ever, in last 12 months.

Had diarrhea----Is this related to the child or the mother? Also is there a timeframe for this? .i.e. ever, in last 12 months.

**Authors’ response**: Dear Reviewer, thank you so much. As you know, the DHS dataset includes the two symptoms mentioned above, which are directly related to the child, with the duration referring to the last two weeks before the survey was conducted. Additionally, the sex of the household head typically refers to the gender of the primary decision-maker or leader of the participants' household.

9. Is this meant to be 'descriptive statistics such as frequencies that are presented as percentages' or are there mutliple techniques here that need further clarification? Try to avoid suggestive words such as 'like', as this implies more was done that is not detailed here. Cleaning and editing or editing and recoding can also be combined as one technique here.

**Authors’ response:** Dear esteemed reviewer, we appreciate your valuable suggestion and we have revised our manuscript as per your recommendation. Kindly have a look at over the revised manuscript.

10. This approach relaxes the assumption of a Gaussian distribution of the observed semi-variogram in the input data, which is often not realistic in practice. A citation could be added here to give weight to this point. GWR and MGWR. Explain these abbreviations first, not sure what they are.

**Authors’ response**: Dear esteemed reviewer, we sincerely apologize for the inconsistencies in our previous submission. We have revised and corrected these issues accordingly. Please refer to the updated manuscript for the changes made.

11. A total weighted of 2562 children aged 6–23 months were included in this study. More than one third (34.5%) of the female children were had taken foods rich in micronutrient. Word not needed here, or meant to be population? Remove 'were'. Think these should come before the statistics on micronutrients to set the scene more on the demographics of the population.

**Authors’ response**: We appreciate your suggestions. We have revised it accordingly.

12. Some of these factors (i.e had diarrhea and cough) are not mentioned at all again in the manuscript. Were they tested for and not significant? Are they adding anything to this analysis? Adequate or inadequate MN prevalence? Please specify the prevalence.

**Authors’ response**: Dear esteemed reviewer, we thank you for the comment. These two variables were tested in the OLS model but not significant. The prevalence described in the manuscript is adequate MN intake and we have added it accordingly.

13. What is the unit of measurement for proportion of micronutrient intake shown in Figure 1? Is this a % of children in this region that meet the adequate level?

**Authors’ response**: Dear esteemed reviewer, we apologize for the lack of clarity. Yes, percentage (%) is the unit of measurement for adequate MN intake across different regions.

14. Hot spot analysis--Repeating methods here, tie into what the results are more. Spatial interpolation---Did it increase or was the highest predictive prevalence just shown here?

**Authors’ response**: Dear esteemed reviewer, we have revised the hot spot analysis as per your suggestion and we thank you for your query regarding spatial interpolation. The results indicate that the highest predictive prevalence of micronutrient intake did indeed increase in the Dire Dawa, Harari, Addis Ababa, Somali, Gambella, and some parts of the Afar regions. In contrast, lower predictive prevalence was observed in the remaining areas of Ethiopia.

15. Sat scan stastical analysis--Why is primary in brackets? Sentence doesn't make sense at the moment. Also what makes them primary/secondary or why most likely? What finding allows you to infer this? Children 'who' were and does window mean the same as cluster or is this something different like a time period?

**Authors’ response**: Dear esteemed reviewer, thank you for your insightful questions. We have revised to better clearly understand by the reader. In spatial scan analysis, a "window" typically refers to a specific geographical area or a defined spatial region used to analyze the distribution of events or characteristics within a dataset. This window can be fixed or variable in size and shape, depending on the analysis goals.

Key points about the window in spatial scan analysis:

- **Geographical Scope**: The window defines the area over which the analysis is conducted, helping to identify clusters of events (e.g., disease occurrences, resource usage).
- **Temporal Aspect**: In some contexts, a window may also refer to a time period during which data is examined, allowing for the identification of trends over time.
- **Clustering**: The window helps determine the statistical significance of clusters, assessing whether the observed concentration of events within that area is greater than expected by chance.

16. Table 3---Is there as reason this number is the opposite way aligned to the others in the table?

**Authors’ response**: Dear esteemed reviewer, we thank you and appreciate your feedback. This presentation does not convey any specific meaning; rather, we have organized it in this way to ensure the best fit and proper utilization of the table.

17. OLS---Positive, negative or a mixture here? Which ones??

**Authors’ response**: Dear respected reviewer, we thank you for your concern. It is a positive association and we have revised it accordingly. Kindly look at the revision made in the revised manuscript.

18. Table 4----Do the double asterix denote significance? Please add this as a note to the table. GWR--Be consistent with abbreviations. The difference was 17, implying... or the difference of 17 implied... This sentence needs further clarification, possibly breaking into two sentences.

**Authors’ response**: Dear respected reviewer, we appreciate your helpful comments. We have added a note in table 4 and we have revised the sentence to better understand by the reader. Thank you so much for your insightful suggestions.

19. MGWR—remove in. This would make more sense as 'additionally' or 'furthermore' as additional rather than contradictory results.

-Sentence doesn't quite make sense, this first part could read 'children from a household that is in the rich wealth index had a probability...'

-Should this read IN the northern eastern...? Or is this a separate factor as the beginning of this sentence states that all other factors are held constant?

-Is this Map D?

**Authors’ response**: Dear esteemed reviewer, we thank you for your comments. We have accepted all the comments and revised the manuscript thoroughly. Kindly see the revision made in the revised manuscript.

20. Discussion---Higher than a study done in Bangladesh and SA, or higher than national data shows from a population survey? Be more specific here. Why would some of these factors such as culture, beliefs, and religious differences make a difference? Do they eat different foods that are or are not rich in Vitamin A or other micronutrients?

**Authors’ response**: Dear esteemed reviewer, we appreciate your concern. Cultural, belief, and religious differences can significantly impact dietary choices and nutritional intake in several ways:

**Cultural Influences**

- **Traditional Foods**: Different cultures have unique culinary traditions that dictate what foods are commonly consumed. These traditions often include specific ingredients that may or may not be rich in vital nutrients like Vitamin A.
- **Food Preparation Methods**: Cultural practices influence how food is prepared. For example, some cooking methods may preserve or destroy micronutrients.

**Beliefs and Values**

- **Dietary Restrictions**: Personal or cultural beliefs can lead to dietary restrictions (e.g., vegetarianism, veganism) that affect nutrient intake. For instance, those avoiding animal products may have lower Vitamin A intake unless they consume fortified foods or supplements.
- **Health Perceptions**: Beliefs about health and nutrition can dictate food choices. Some cultures may prioritize certain foods for their perceived health benefits, affecting the overall nutrient profile of their diet.

**Religious Differences**

- **Fasting and Feasting**: Many religions have specific periods of fasting or feasting that can alter dietary patterns. For example, during Ramadan, Muslims may consume different foods at specific times, influencing their overall micronutrient intake.
- **Prohibited Foods**: Religious laws may restrict certain foods (e.g., pork in Islam and Judaism), which can impact the diversity of the diet and the intake of essential nutrients.

**Dietary Implications**

- **Vitamin A Sources**: Foods rich in Vitamin A (e.g., liver, fish, dairy products, and certain fruits and vegetables) may not be as prevalent in some diets. For instance, cultures that primarily consume plant-based diets may rely more on carotenoid-rich foods (like carrots and sweet potatoes) for Vitamin A.
- **Nutritional Education**: Awareness of micronutrient needs varies by culture, which can lead to differences in how individuals prioritize nutrient-rich foods.

In summary, cultural, belief, and religious factors play crucial roles in shaping dietary practices, which directly impacts the intake of essential nutrients like Vitamin A. Understanding these influences is vital for addressing nutritional deficiencies in diverse populations.

21. Citation could be added here. Also, as wealth index was also tested, could you say that most of those with a higher index lived in these areas? Therefore, tying together your point below about purchasing power too in one place. Also, can then differentiate that it is not because of the location, but the income. 'Access to a diverse range of nutrients' or similar would make more sense here. Animal-based is the correct term here. Significant predictors of micronutrient intake? Positively or negatively? Lots of citations available for this, especially region specific.

**Authors’ response**: Dear esteemed reviewer we thank you for your helpful suggestion. We have revised the manuscript in light of your vital suggestion. Thank you once again for your insightful recommendation.

22. Please explain further how inadequate sanitation leads to inadequate intake? Is this to do with health outcomes or because of a lack of suitable food prep environment etc?

**Authors’ response**: Dear respected reviewer thank you for your important comment. Inadequate sanitation can significantly impact nutritional intake and overall health through several interconnected mechanisms:

**Health Outcomes**

1. **Increased Illness**: Poor sanitation often leads to waterborne diseases (e.g., cholera, dysentery) and other infections. When individuals are frequently ill, their bodies may not absorb nutrients efficiently, leading to malnutrition.
2. **Nutritional Deficiencies**: Chronic infections can increase nutrient requirements and affect appetite. This may result in inadequate intake of essential nutrients, exacerbating health issues.

**Food Preparation Environment**

1. **Contamination Risks**: Inadequate sanitation can lead to contamination of food and water supplies with pathogens. This makes it risky to consume food that may otherwise be nutritious, discouraging intake of potentially healthful foods.
2. **Limited Access to Clean Water**: Clean water is essential for food preparation and hygiene. A lack of it can hinder safe cooking practices, leading to reliance on unsafe food sources or improperly prepared meals.

**Socioeconomic Factors**

1. **Economic Burden**: Health problems resulting from poor sanitation can lead to increased healthcare costs and lost productivity, making it difficult for families to afford and access nutritious foods.
2. **Food Security**: In areas with inadequate sanitation, agricultural productivity may decline due to disease and environmental degradation, limiting the availability of safe, nutrient-rich foods.

**Behavioral Changes**

1. **Avoidance of Certain Foods**: Individuals may avoid certain foods due to fear of contamination, leading to a less diverse diet and inadequate intake of essential nutrients.
2. **Coping Strategies**: In response to health issues caused by poor sanitation, individuals may resort to less nutritious, convenience foods that are easier to prepare but lack essential micronutrients.

In summary, inadequate sanitation creates a cycle of health issues and food safety concerns that hinder proper nutrition. Addressing sanitation is crucial not just for immediate health outcomes but also for ensuring a safe and conducive environment for food preparation and consumption.

23. Child age-discussion section--Clarify if this is meant to be incongruent and so the results do not align with other studies, or this result is congruent with other studies. Due to encouragement of continuation of breastfeeding or because of another reason?

**Authors’ response:** Dear respected reviewer thank you so much for your comment. In the discussion regarding child age and its impact on nutrition, it’s essential to clarify whether the results are incongruent or congruent with existing studies. Here’s how to approach this:

**Clarification of Results**

1. **Congruent Findings**: If the results align with other studies, this could suggest a consistent understanding of child nutrition across various contexts. This alignment might be due to:
   - **Encouragement of Breastfeeding**: Many studies emphasize the importance of breastfeeding during infancy and toddlerhood, which has well-documented benefits for child health and nutrition.
   - **Nutritional Guidelines**: Consistency in guidelines promoting age-appropriate complementary feeding after breastfeeding may also contribute to similar findings in various studies.
2. **Incongruent Findings**: If the results do not align with previous studies, it may indicate:
   - **Variability in Practices**: Differences in cultural practices, health policies, or community support for breastfeeding could lead to divergent outcomes.
   - **Sample Characteristics**: Variations in study populations (e.g., socioeconomic status, geographic location) may yield different results regarding child age and nutrition.
   - **Methodological Differences**: Differences in data collection methods or definitions of age categories may also result in incongruences.

**Possible Reasons for Results**

- If results are congruent, it may reflect a broader acceptance of breastfeeding practices and a deeper understanding of child nutritional needs.
- If results are incongruent, further investigation is warranted to explore contributing factors, such as local health initiatives, educational programs, or societal attitudes toward breastfeeding and complementary feeding.

In conclusion, clearly stating whether the results are congruent or incongruent with other studies allows for a more nuanced discussion about child age and nutrition. It also helps identify the underlying reasons for these findings, whether they stem from breastfeeding practices, cultural influences, or methodological differences.

24. Job or work discussion better to correct study as studies and "The possible justification may be that mothers who are employed have greater financial resources to purchase a diverse range of nutrient-rich foods for their families" - better wording.

**Authors’ response**: Dear esteemed reviewer thank you for your crucial suggestions. We have accepted and corrected it accordingly.

25. Could this also be because mothers who are in employment are possibly more educated and so are more knowledgeable about nutrition?

**Authors’ response**: Dear esteemed reviewer thank you for raising such an important question. Yes, the observation that employed mothers may have better nutritional outcomes for their children can be attributed to several factors, including education and knowledge about nutrition. Here are some key points to consider:

**Education and Knowledge**

1. **Higher Educational Attainment**: Employed mothers often have higher levels of education, which is associated with greater awareness of nutritional guidelines and health practices. This can lead to more informed choices regarding their children’s diets.
2. **Access to Nutritional Information**: Educated individuals are generally more adept at seeking out and understanding nutritional information, leading to improved dietary practices for themselves and their families.

**Economic Resources**

1. **Increased Financial Stability**: Employment provides a source of income, allowing mothers to afford healthier food options, such as fresh fruits, vegetables, and whole grains, which are essential for optimal child nutrition.
2. **Access to Healthcare**: Employed mothers may have better access to healthcare services, including prenatal and postnatal care, which can positively impact maternal and child health outcomes.

**Social and Environmental Factors**

1. **Support Networks**: Employed mothers may be more likely to engage with social networks and community resources that promote healthy eating and nutrition education.
2. **Time Management Skills**: Employment may encourage better time management practices, enabling mothers to plan and prepare nutritious meals more effectively.

In summary, the relationship between maternal employment and child nutrition can indeed be influenced by the education level and nutritional knowledge of mothers. This connection highlights the importance of supporting educational and employment opportunities for women as a means to improve child health and nutrition outcomes.

26. Strength and limitation section----Could also be a limitation that the adequacy of MN intake was measured as a binary variable, with a continuous outcome, more detailed analysis could be conducted as to if children are meeting the daily requirement of the most important MNs, or where this differs on a scale across regions or when in combination with other factors.

**Authors’ response**: Dear esteemed reviewer, we thank you for this critical issue. Indeed, the measurement of micronutrient (MN) intake as a binary variable presents both strengths and limitations in evaluating nutritional adequacy. Here’s how to address this in the strengths and limitations section:

**Strengths**

- **Simplicity**: Using a binary variable allows for straightforward interpretation of whether children meet or do not meet the minimum micronutrient intake. This can facilitate quick assessments and comparisons across populations.
- **Clear Thresholds**: Binary categorization helps in identifying clear cutoffs for public health interventions, making it easier to communicate findings to stakeholders.

**Limitations**

1. **Loss of Nuance**: By categorizing MN intake as binary, the analysis overlooks the nuances of actual nutrient consumption. It does not account for varying degrees of adequacy, which could provide deeper insights into dietary patterns.
2. **Continuous Outcomes**: A continuous measure of MN intake would allow for more detailed analysis, enabling researchers to:
   - Assess whether children are meeting specific daily requirements for critical micronutrients.
   - Identify variations in intake levels across different regions, socioeconomic statuses, or demographic groups.
   - Explore the interplay between multiple factors (e.g., dietary diversity, health status) and their impact on micronutrient adequacy.
3. **Regional and Contextual Differences**: A more granular analysis could reveal important disparities in micronutrient intake based on geographic location or cultural dietary practices, which binary measures may obscure.

**Recommendations**

- Future studies should consider employing continuous measures of micronutrient intake to capture the complexity of dietary adequacy. This would enhance understanding of nutritional status and inform targeted interventions more effectively.

By acknowledging these limitations, the research can be positioned to advocate for more comprehensive approaches in future studies, ultimately leading to improved public health strategies.

**Reviewer #4:**

I wanted to bring to your attention that I previously reviewed manuscript “PONE-D-24-22661” and provided several comments and suggestions for revision. However, in the current version, I do not see any changes that indicate my feedback was addressed. If the revisions have been made, I would appreciate receiving a version with the changes clearly highlighted or tracked.
**Authors’ response**: Dear esteemed reviewer, we are unsure about your reference, as we received the manuscript comments on October 4, 2024. Unlike the other reviewers, we have not yet received your feedback; it simply states that Reviewer 4 has not submitted comments. We sincerely apologize for any confusion this may have caused, and we recommend reaching out to the academic editor for further clarification.

**Reviewer#5**

This relevant work aimed to assess the spatial distribution and factors influencing the intake of foods rich in MN among children aged 6–23 months in Ethiopia. The study has national representativeness and a robust statistical analysis.

Below are some minor and major comments:

**Minor comments:**

1. The text does not define the meaning of ANC, nor how the wealth index variable is constructed

**Authors’ response**: Dear esteemed reviewer, we apologize for the inconsistency. We have written out the full term for ANC as it first appears in the text. Additionally, we have re-categorized the five-scale wealth index in to three category as "poorest" and "poor" have been combined as "poor," "middle" has been changed to "medium," and "rich" and "richest" have been categorized as "rich."

2. Regarding table 1:

Why not evaluate age as a continuous variable?

Sex of the household? (word “head” is missing)

Family size is repeated

**Authors’ response**: Dear we thank you for your insightful question. We chose to categorize age into discrete groups in our analysis to simplify interpretation and enhance the clarity of our findings. This approach allows us to focus on specific age ranges that may exhibit distinct patterns or trends relevant to our study objectives (spatial variation across age groups). We have corrected the missing word and delete the repeated word. Kindly review the revised manuscript.

3. To include AIC in the second part of Table 4? If I understand correctly, the AIC is a criterion to select between more than one model, what would be the use of adding it in isolation?

**Authors’ response**: Dear Reviewer, Yes, you are correct that AIC is used for model evaluation. However, the authors primarily intended to report the AIC value of OLS in Table 4, and this will be repeated in Table 5 when there is a comparison of three models. If it is repeated, we can certainly delete it from Table 4. Thank you so much for your suggestion.

**Major comments:**

4. Some variables of nutritional status in the infant. Is it correct to assume that infants who have not consumed MN in the last 24 hours have MN deficiency? I think that is not an adequate assumption, it could be possible that they do not have MN deficiency even if they have not consumed MN in the previous 24 hours. Did you take any consideration regarding breastfeeding? Breastfeeding could also provide NM to the infant. If you do not have access to this information, you could at least develop this point in the discussion.

**Authors’ response**: Dear Reviewer, we thank you for your insightful comments. We acknowledge that this assumption may not adequately reflect the nutritional status of all infants, as they could receive essential nutrients through breastfeeding.

In our study, we didn’t consider the potential impact of breastfeeding on MN intake since we did not have comprehensive data on breastfeeding practices among the participants. We appreciate your suggestion to address this limitation in the discussion section, and we have included it as a limitation as the data source was secondary and a more detailed explanation of how breastfeeding may contribute to MN levels in infants, even in the absence of recent MN consumption. Thank you for your valuable feedback, which will help enhance the quality of our manuscript.

5. Nutrient intake was assessed by 24-hour recall, and the day of the week was indicated. If this survey was conducted on a Monday, it is possible that the previous day is not representative of usual dietary behaviour. This is an important limitation that should be addressed in the discussion.

**Authors’ response**: Dear reviewer, we appreciate your insight to enhance the manuscript quality. We have accepted and revised it accordingly as per your suggestion.

6. I think the discussion could include something about the validation of the method to assess NM intake, perhaps how close the questionnaire is to the gold standard (serum measurement) this argument would give strength to the discussion.

**Authors’ response**: Dear esteemed reviewer, we thank you for your valuable suggestion. We agree that discussing the validation of our method for assessing nutrient intake is important. We have included information on how closely our questionnaire aligns with the gold standard of serum measurement. This comparison will strengthen our discussion and provide a clearer context for the reliability of our findings. We have revised it according to your vital suggestion.

7. The discussion is developed around the purchasing power of families and therefore the ability to acquire foods rich in NM. However, there is a lack of information about the food environment in the centre of urban areas, and how this could be promoting or not the consumption of foods rich in NM (commercial determinants of nutrition).

**Authors’ response**: Dear reviewer, we thank you so much for your issue. Since we have used the Ethiopian Demographic and Health Survey (EDHS) that provides valuable data on demographic and health indicators, it may not comprehensively capture the specific nuances of the food environment or the commercial determinants of nutrition in urban settings.

To address this gap, we acknowledge the importance of incorporating qualitative and quantitative data that reflect the local food landscape, including availability, accessibility, and marketing of nutrient-rich foods. We recommend the future researcher to explore additional secondary data sources that focus on urban food environments, such as local market surveys or studies specifically examining food access and dietary patterns in urban Ethiopian contexts.

In future iterations of our discussion, we have highlighted the limitations of relying solely on purchasing power without considering the broader food environment, and we have emphasized the need for more targeted research to fully understand how these factors interact. This will strengthen our argument and provide a more comprehensive analysis of the barriers and facilitators to accessing nutrient-rich foods in urban areas. Thank you for guiding us to enhance the depth of our discussion.

8. I think the study includes many results and some of them evaluate the same associations, not all the results are discussed, you should consider sending some to supplementary material. You could mention in the introduction or discussion how spatial distribution studies have been useful in planning public health and nutrition intervention projects.

**Authors’ response**: Dear esteemed reviewer, we thank you for the comment and suggestion. We have accepted and revised it accordingly. Kindly review the revised version of the manuscript.

9. An important issue to consider in the discussion is the possible reverse causality in children who consume MN supplements since they usually do so when they already have a diagnosis of deficiency.

**Authors’ response**: Dear esteemed reviewer, we appreciate your insightful comment regarding the potential for reverse causality in children consuming micronutrient (MN) supplements. We acknowledge that supplementation often occurs in response to a diagnosed deficiency, which can complicate our analysis of the relationship between MN intake and health outcomes.

To address this concern, we have clarified in the discussion that the observed effects may not solely stem from supplementation but rather from pre-existing health conditions that necessitate it. We have also explored the implications of this reverse causality on our findings and highlight the importance of longitudinal studies to better establish cause-and-effect relationships in future research. Thank you for bringing this critical issue to our attention.
